# Enhancing *CYP2D6* genotyping with nanopore sequencing to address allele diversity *P. vivax* malaria elimination

**DOI:** 10.1038/s41598-025-05065-2

**Published:** 2025-09-30

**Authors:** Thidathip Wongsurawat, Piroon Jenjaroenpun, Kanokon Suwannasin, Natnicha Wankaew, Naphat Sanguansakpakdee, Thananya Amornpornviwat, Arjen M. Dondorp, Nicholas P. J. Day, Francois Nosten, Nicholas J. White, Mallika Imwong

**Affiliations:** 1 Siriraj Long-Read Lab, Oxford Nanopore Centre of Excellence, Division of Medical Bioinformatics, Research Department, Faculty of Medicine Siriraj Hospital, Mahidol University, Bangkok, 10700 Thailand; 2 Department of Biomedical Informatics, College of Medicine, University of Arkansas for Medical Sciences, Little Rock, AR 72205 USA; 3 Department of Pharmacology, Faculty of Medicine Siriraj Hospital, Mahidol University, Bangkok, 10700 Thailand; 4 Mahidol-Oxford Tropical Medicine Research Unit, Faculty of Tropical Medicine, Mahidol University, Bangkok, Thailand; 5 Centre for Tropical Medicine and Global Health, Nuffield Department of Medicine, University of Oxford, Oxford, UK; 6 Shoklo Malaria Research Unit (SMRU), Faculty of Tropical Medicine, Mahidol-Oxford Tropical Medicine Research Unit, Mahidol University Mae Sot, Bangkok, Thailand; 7 Department of Molecular Tropical Medicine and Genetics, Faculty of Tropical Medicine, Mahidol University, Bangkok, Thailand

**Keywords:** Thai, Long-read sequencing, Turnaround time, Pharmacogenomics, Genetics, Sequencing, Next-generation sequencing

## Abstract

The precise profiling of *CYP2D6* alleles is critical for identifying patients who are likely to benefit from primaquine therapy, a standard treatment for preventing *P. vivax* relapse and, as a result, obstacle for malaria elimination. Conventional assays often fall short, as they do not capture the wide range of allele diversity found at the *CYP2D6* locus, particularly in populations from malaria-endemic regions. In response, we developed a methodology integrating cost-effective DNA library preparation with nanopore sequencing, reducing reliance on extensive third-party enzymes and reagents and shortening processing times to enhance accessibility. Using well-characterized DNA standards, we evaluated various bioinformatics tools for their ability to accurately call *CYP2D6* star alleles. Nanopore sequencing proved particularly valuable for identifying complex structural variants, which led us to propose a novel approach to manage hybrid structural variations using long-read amplicon data. We conducted an in-depth genotypic analysis of DNA samples from 90 individuals in Thailand’s Greater Mekong Subregion, achieving a high level of resolution in *CYP2D6* genotyping. This analysis not only identified structural variants but also a previously unidentified allele. The findings suggest potential paths forward for testing that is sensitive to local genetic variability and supports informed policy decision-making in real time, thereby potentially enhancing the effectiveness of *P. vivax* elimination.

## Introduction

Primaquine (8-aminoquinoline), a World Health Organization-approved antimalarial medicine, has limited schizonticidal action against the blood stages of *Plasmodium vivax* malaria, but its principal efficacy is in targeting hypnozoites, the parasite’s latent liver stage. These hypnozoites can induce malaria relapses months or even years after the initial infection, thus adequate treatment is essential for preventing subsequent episodes. According to the current WHO guidelines, the recommended primaquine treatment regimen consists of a 14-day course at a dosage of 0.25–0.5 mg/kg/day. The specific dosage depends on the geographic origin of the patient, as variations in drug response and the prevalence of certain strains may influence treatment efficacy^1^. These guidelines aim to ensure that patients receive optimal care while minimizing the risk of relapse and further transmission of the disease.

By effectively eliminating hypnozoites, primaquine plays a vital role in malaria management strategies, particularly in regions where *P. vivax* is endemic^2,3^. This approach not only helps to control malaria cases but also contributes to broader public health efforts to combat malaria resurgence in affected areas. Two metabolic pathways have been identified, comprising the cytochrome P450 CYP2D6 isoenzyme and monoamine oxidase-A (MAO-A). Carboxyprimaquine, the most abundant plasma metabolite, is produced via the MAO-A-mediated pathway but is not considered hypnozoitocidal. The active phenolic metabolites that are produced as a result of metabolism through CYP2D6 are expected to possess anti-malarial properties that are mediated by the production of oxidative stress through redox cycling^4–8^. Studies show that CYP2D6 is crucial for primaquine metabolic activation, and mutations in *CYP2D6* can potentially affect its efficacy^9–14^.

The effectiveness of primaquine is influenced by patient-specific genetic differences in the cytochrome P450 2D6 (CYP2D6) enzyme, which can affect drug metabolism and can vary according to the geographic location and ethnicity of the patient^15^. The *CYP2D6* gene exhibits significant variation that can influence drug efficacy and patient safety. Understanding these genetic differences is crucial for the global fight against malaria, because they can dictate the success or failure of treatment regimens. However, in areas burdened by malaria, the underlying genetic diversity has been largely unexplored due to limited research focus and technological access.

Current methods for assessing the *CYP2D6* genotype, such as single-nucleotide polymorphism (SNP) genotyping, have made strides in identifying common alleles associated with drug metabolism^16^. However, these techniques often fall short in areas with populations that have diverse and unique genetic backgrounds, because the techniques rely on databases of known variants, which may not reflect the full spectrum of genetic diversity. Short-read sequencing detects novel alleles but can struggle with the structural complexity of *CYP2D6*, leading to misalignments and inaccurate variant calls^17^. In addition, the short-read sequencing platform has high capital costs and maintenance costs. A substantial need remains for comprehensive, inexpensive, and inclusive genotyping approaches that can capture the gene’s polymorphic nature in populations with diverse genetic backgrounds.

To address this need, use of nanopore sequencing technology is a promising approach for targeted gene genotyping. It can deliver high-resolution, long-read sequencing data, and its portability and lower setup costs make it particularly appealing for use in remote^18^, resource-limited settings, as well as those often hit hardest by malaria. By facilitating field-based sequencing, this technology could transform *CYP2D6* genotyping globally, offering a detailed insight into local genetic variations. Although nanopore sequencing is advantageous for its detailed, long-read capabilities, it is not without challenges. The complexity of the *CYP2D6* gene can make analysis difficult, requiring meticulous data interpretation.

To take advantage of the nanopore technology, our investigation was methodically segmented into 2 distinct phases. In the first phase, we used 12 well-characterized standard DNA samples and 12 clinical DNA samples with known *CYP2D6* genotypes to validate our new integrated wet lab protocol, as successfully performed in HLA-class I typing^18^, and accompanying commonly used star allele identification tools. In the second phase, we extend the validated methods from Phase 1 to a broader cohort consisting of 90 DNA samples collected from the Greater Mekong Subregion in Thailand, enabling us to calculate allele frequency and to manually validate new variants and structural variants. Results of this study offer an alternative method of in-field delivery of *CYP2D6* genotype data, presenting a unique opportunity for point-of-care diagnostics and immediate clinical decision-making.

## Materials and methods

### Study design and DNA samples

**Fig. 1:**
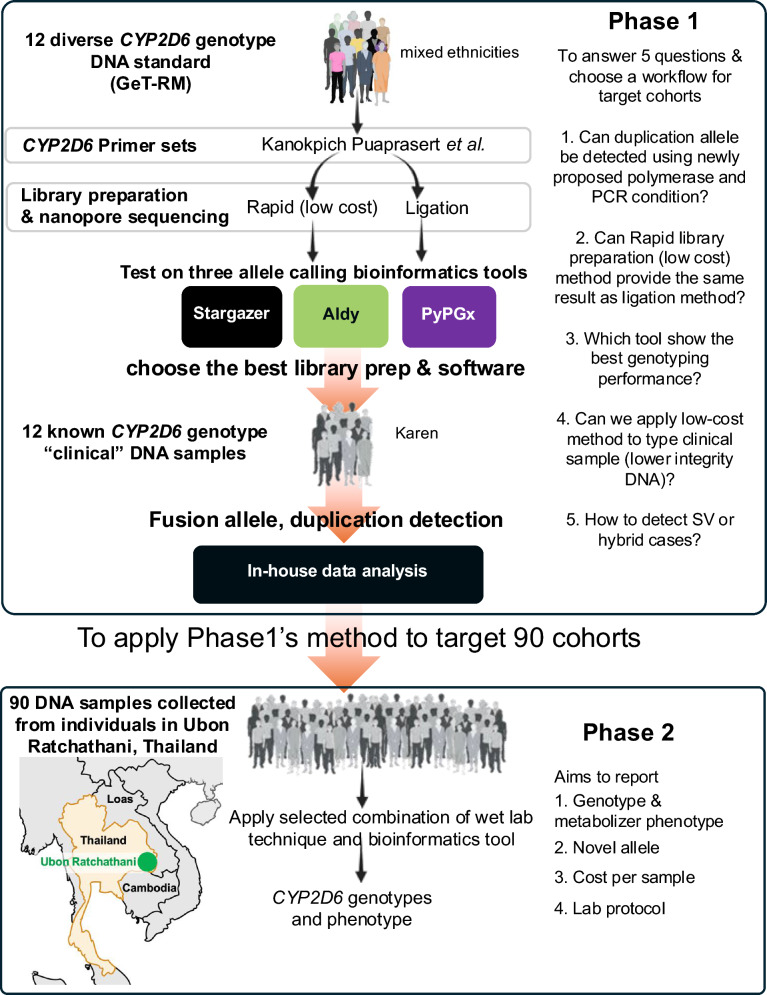
Overall study design. A structured research divided into two distinct phases, focused on the analysis of *CYP2D6* genotypes in a diverse population. Initially, Phase 1 commences with the evaluation of 12 varied *CYP2D6* genotypes derived from the Coriell Institute, representing a mix of ethnic backgrounds. The genotypes are analyzed using standard DNA and several primer sets designed by Kanokpich Puaprasert et al*.*^19^ This stage explores two library preparation techniques for nanopore sequencing: Rapid approach (cost-effective method) and Ligation approach. The effectiveness of these techniques is assessed through three allele-calling bioinformatics tools: Stargazer^20^, Aldy^21^, and PyPGx^22^, with the aim of selecting the optimal library preparation and software for further analysis. Additionally, individual case reviews of known *CYP2D6* genotypes are conducted using the Integrative Genomics Viewer (IGV) to facilitate in-house data scrutiny. Phase 2 extends the validated methods from Phase 1 to a broader cohort consisting of 90 DNA samples collected from individuals in Ubon Ratchathani, Thailand. The objectives of this phase include detailed reporting on the genotype and metabolizer phenotype of the samples, the identification of novel alleles, and a comprehensive evaluation of the cost-effectiveness per sample and the overall lab protocol. This structured approach ensures systematic data collection and analysis, aiming to enhance understanding of *CYP2D6* genetic variability across different populations. SV: Structural Variation.

The study design is shown in Fig. 1. Three sets of DNA samples used in this study. First set is 12 well-characterized DNA standard samples obtained from GeT-RM (Coriell Institute for Medical Research, USA; https://www.coriell.org/), a collection that has been studied for pharmacogenomics with available data on *CYP2D6* gene patterns from various testing methods. The primer set was investigated with 2 methods for library preparation: rapid library preparation and ligation library preparation.

The second set of samples comprised twelve blood specimens collected from the Karen population living in Tak, one of the western provinces of Thailand, which had previously undergone qPCR analysis. The third set included 90 Thai DNA samples from Ubon Ratchathani, located in northeastern Thailand. The written informed consent from all participants was conducted. The collection, processing, and handling of venous blood samples adhered to the Appropriate Technology in Health (PATH) guidelines. The study received ethical approval from the review committees of the Faculty of Tropical Medicine at Mahidol University (approval no. MUTM2012-045-12, MUTM 2012-068-11, MU2019-025). This ensures that all experiments adhered to relevant guidelines and regulations, with informed consent obtained from all participants, and were conducted in accordance with the Declaration of Helsinki^23^.

An EDTA-anticoagulated blood sample was drawn before the initiation of therapy and stored at − 20 °C until DNA extraction at the Faculty of Tropical Medicine, Mahidol University, Bangkok. DNA purification was performed using a QIAgen kit (QIAgen, Germany) in accordance with the manufacturer’s instructions. The isolated genomic DNA samples were then stored at 4 °C until further processing.

### Long-range PCR and primer sets

*CYP2D6*-specific primers were previously reported by Kanokpich Puaprasert et al. Primer 1.1 (2DPKup, 5ʹ-GTTATCCCAGAAGGCTTTGCAGGCTTCA-3ʹ) and Primer 1.2 (2DPKlow, 5ʹ-GCCGACTGAGCCCTGGGAGGTAGGTA-3ʹ) span the full *CYP2D6* gene, aiming for a 5.1-kb product. Primer 2.1 (2D6dupl-F, 5ʹ-CCTGGGAAGGCCCCATGGAAG-3ʹ) and Primer 2.2 (2D6dupl-R, 5ʹ-CAGTTACGGCAGTGGTCAGCT-3ʹ) target the duplication region, for a 3.5-kb product. Finally, Primer 3.1 (5′2D65, 5ʹ-CACCAGGCACCTGTACTCCTC-3ʹ) and Primer 3.2 (3′2D65, 5ʹ-CAGGCATGAGCTAAGGCACCCAGAC-3ʹ) aim for the deleted region, also with a 3.5-kb product.

The PCR reaction mixture (25 μL total volume per reaction) consisted of 5 μl of 5X PrimeSTAR GXL Buffer, 2 μl of dNTP mixture (2.5 mM each), 1 μl each of primers 1.1 and 1.2 (10 μM), and 0.5 μl each of either primers 2.1 and 2.2 or primers 3.1 and 3.2 (10 μM); 1 μl of PrimeSTAR GXL DNA Polymerase and 14 μl of DNA with nuclease-free water also were added. This mixture ensures optimal conditions for amplifying the target DNA segments. PCR conditions were as follows: primary denaturation at 98 °C for 1 min; 32 cycles at 98 °C for 10 s and 68 °C for 45 s; and a hold at 4 °C. The long-range PCR reactions were performed with a T100 thermal cycler (Bio-Rad, USA). Amplified samples were quantified with a Qubit dsDNA HS kit (Applied Biosystems, USA), and PCR products were analyzed with agarose gel electrophoresis (2% agarose).

Rapid library preparation (detailed protocol is in the Supplementary Protocol).

The amplicons were used as input DNA for the Rapid Barcoding Kit (SQK-RBK004 and SQK-RBK110.96; ONT, UK); amplicons were individually barcoded according to the manufacturer’s protocol. The 7.5 µL volume of input DNA in each tube was adjusted to 2.5 µL per sample in 5 µL nuclease-free water. Sequencing was performed on a GridION device for 48 h. Twelve or 96 samples were combined per R9.4.1 flow cell (FLO-MIN106D; ONT, UK).

### Ligation library preparation

The amplicons were used as input DNA for the Native Barcoding Kit (EXP-NBD104; ONT, UK) combined with the Ligation Sequencing Kit (SQK-LSK109; ONT, UK); amplicons were individually barcoded according to the Native Barcoding Amplicons protocol. The amount of input DNA in each tube was adjusted to 100 ng per sample in 48 µL nuclease-free water. Sequencing was performed on a GridION device for 48 h.

### Nanopore sequencing, data preprocessing, and bioinformatics analysis

Nanopore sequencing was performed with MinKNOW software, version (v22.05.8), which collected read data in the form of fast5 files. The reads were converted into base sequences and demultiplex with the super-accuracy model (SUP) of the Guppy base-caller (v5.0.16). The quality of the sequences was assessed with NanoPlot (v1.41.0), and Porechop (v0.2.4) was used to trim any remaining adapter sequences and barcodes in the samples. The resulting Fastq files were used as input for Minimap2 (v2.28) to map the sequences to the human genome (version hg19) with the parameters ‘-ax map-ont -secondary = no’. The outputs in BAM format then were then processed to identify single-nucleotide variants with Clair3 (v1.0.7) with the phasing function disabled, resulting in variant call format (VCF) files. the Integrative Genomics Viewer (IGV) (v2.14.1) was used for sequence visualization.

### Star allele identification

To identify *CYP2D6* star alleles, we analyzed sequencing data generated from both Rapid and Ligation library preparations. We utilized DNA standard samples obtained from the Genetic Testing Reference Material (GeT-RM) program, which contained known *CYP2D6* star alleles. These reference samples were used to validate and compare the performance of three allele-calling bioinformatics tools: Stargazer (v2.0.0)^20^, Aldy (v4.6)^21^, and the PyPGx package (v0.25)^22^, available at https://github.com/sbslee/pypgx. Each tool was run using the variant call format (VCF) files generated by Clair3 (v1.0.7) from the preprocessing step.

For Aldy, the parameters ‘-p pacbio-hifi-targeted’, ‘-g cyp2d6’, and ‘-genome hg19’ were specified. For Stargazer, the parameters ‘-t cyp2d6’ and ‘-a hg19’ were used. For PyPGx, the ‘run-long-read-pipeline’ command was executed with ‘CYP2D6’, along with the parameters ‘-assembly GRCh37’ and ‘-force’. All other settings were left at their respective default values.

The results were cross-validated using the GeT-RM reference samples, and any discrepancies in allele calls were visualized in the IGV to inspect the sequencing reads and ensure accuracy.

### In-house data analysis

Upon obtaining nanopore sequencing reads, we mapped them to the human reference genome (hg19) to detect standard and complex variations, including duplications, deletions, and hybrid structures. Reads were classified into four groups: (1) those with no hybrid (no_hybrid), (2) CYP2D6-D7 hybrid intron1 (hybrid_intron1), (3) *CYP2D6-D7* hybrid between intron2 and exon8 (hybrid_intron2_exon8), and (4) CYP2D6-D7 hybrid exon 9 (hybrid_exon9).

Single-nucleotide variant (SNV) calling was performed using Clair3 (v1.0.7) with the r941_prom_sup_g5014 model^24^, and phasing was done with WhatsHap (v2.3)^25^. Each of the four read groups was processed separately for SNV detection, enabling accurate haplotype-specific SNP identification. After variant calling, consensus sequences were generated using Flye polishing (v2.9.3)^26^, followed by additional polishing with Medaka (v1.11.3) using the r941_min_sup_g507 model.

For validation, known key SNPs were compared against our consensus sequences using BLAST, and any mismatches were manually curated to confirm their validity. This workflow allowed for the assignment of diplotypes, providing a comprehensive analysis of the functional status of the CYP2D6 gene, as outlined in our in-house data analysis workflow (Supplementary Fig. 1). Selected outputs were further validated and visualized using IGV for graphical inspection and review.

### Translating of star alleles into metabolizer profiles

Metabolizer profiles are determined based on activity scores derived from *CYP2D6* diplotypes, following the guidelines set by the Clinical Pharmacogenetics Implementation Consortium (CPIC). The activity score of a *CYP2D6* diplotype is calculated by summing the values assigned to each allele. For *CYP2D6* alleles with variable copy numbers, the activity value of an allele is multiplied by the number of gene copies. According to CPIC guidelines, metabolizer phenotypes are classified as follows: individuals with an AS of 0 are poor metabolizers (PMs), those with an AS between 0 and 1.25 are intermediate metabolizers (IMs), scores from 1.25 to 2.25 indicate normal metabolizers (NMs), and scores above 2.25 signify ultrarapid metabolizers (UMs). All the plots were created in R (Version 1.4.1103).

## Results

### Establishing experimental and bioinformatics workflow

**Fig. 2:**
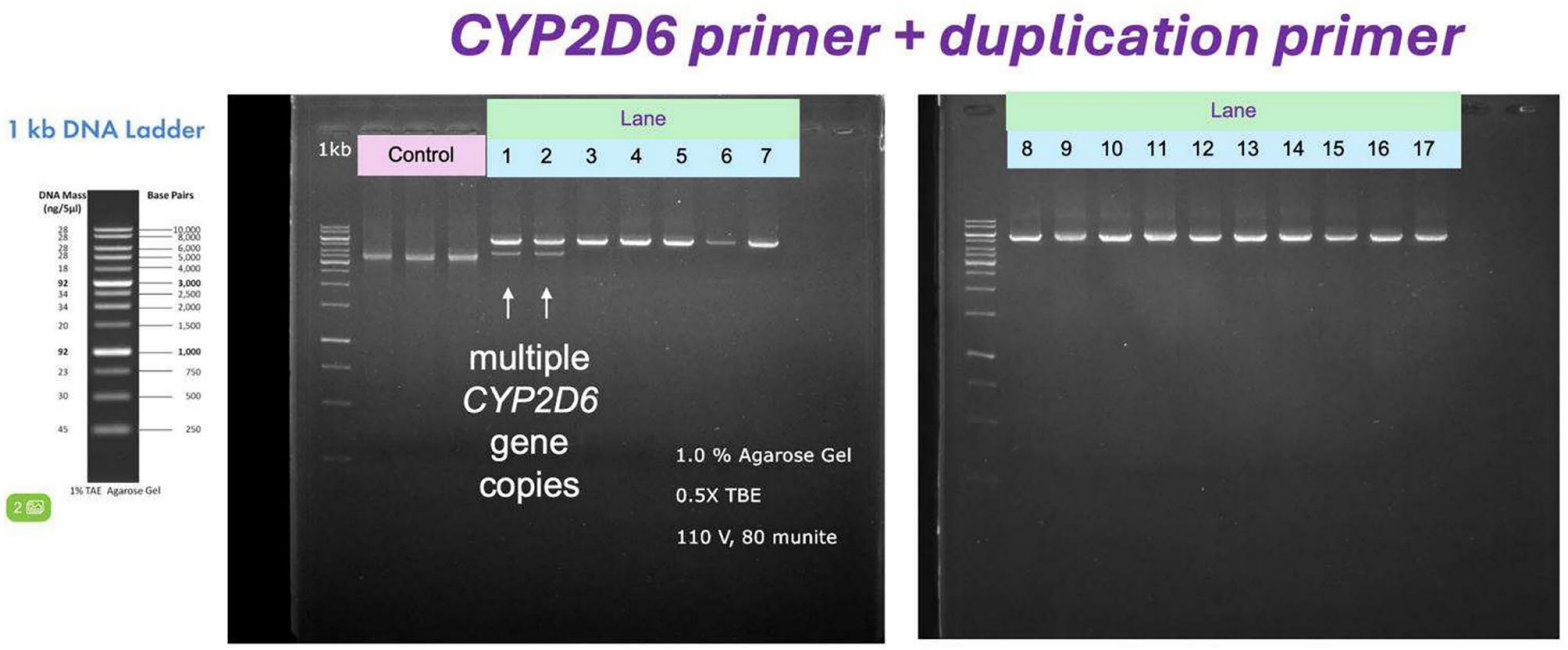
Whole *CYP2D6* gene (~ 5.1 Kb) and duplication region amplification (3.5 Kb). Lanes 1 and 2 present the results of PCR amplification of DNA from samples with more than 1 copy of *CYP2D6*. Lanes 3–12 display the outcomes of PCR amplification of DNA from samples that have only a single of *CYP2D6* gene.

The goal of phase 1 was to establish a workflow for phase 2, including experimental and bioinformatics parts, for analyzing the *CYP2D6* gene. The first objective was to identify PCR conditions that not only amplify the *CYP2D6* gene for sequencing but also indicate gene duplications. This study used a primer set published by Kanokpich Puaprasert et al.^19^ but we modified the polymerase, PCR conditions, and methods to enhance genotyping efficiency. As expected, we amplified the whole *CYP2D6* gene as well as its duplication region in the samples that have more than one *CYP2D6* (Fig. 2).

The PCR products of 12 samples were used to prepare libraries and to sequence the DNA with nanopore technology. For library preparation, 2 methods were used—Rapid library preparation and Ligation library preparation; the Rapid method is less expensive, less complex, less time consuming than the Ligation method. To identify the optimal method for library preparation, we determined the predictive performance, or F1 score, of both methods. Overall, the Rapid library preparation method resulted in higher F1 scores than the Ligation library preparation method (Table 1).

**Table 1:** Genotyping results and F1 score from three bioinformatics tools for twelve standard DNA samples using Rapid and Ligation library preparation methods.

Get-RM ID	Diplotype	Aldy		PyPGx		Stargazer	
		Rapid	Ligation	Rapid	Ligation	Rapid	Ligation
NA02016	*2XN/*17	*1/*1	*1/*1	*2/*17	*2/*2	*2/*17	*2/*2
NA07439	*4XN/*41	*34/*119	*34/*119	*2/*69	*2/*69	*4/*41	*4/*41
NA12244	*35/*41	*1/*119	*1/*119	*2/*41	*2/*41	*35/*41	*35/*41
NA12813	*2/*4	*4J/*34	*4J/*34	*2/*4	*2/*4	*2/*4	*2/*4
NA17039	*2/*17	*1/*1	*1/*1	*2/*17	*2/*17	*2/*17	*2/*17
NA17052	*1/*1	*1/*1	*1/*1	*1/*1	*1/*1	*1/*1	*1/*1
NA17058	*10/*10	*1/*1	*1/*1	*10/*10	*10/*10	*10/*10	*10/*10
NA17119	*1/*2	*1/*2	*1/*2	*1/*2	*1/*2	*1/*2	*1/*2
NA17203	*4/*35	*4J/*34	*4J/*34	*2/*4	*2/*4	*4/*35	*4/*35
NA17240	*1/*10	*1/*10	*1/*10	*2/*10	*2/*10	*1/*10	*1/*10
NA17280	*3/*59	*3/*59	*3/*59	*2/*3	*2/*3	*3/*59	*3/*59
NA23093	*1/*10 + *36	*1/*10	*1/*39	*2/*10	*1/*2	*1/*10	*1/*39
F1 score		0.33	0.33	0.5	0.42	0.92	0.83

Bold highlighting indicates a genotype consistency.

**Fig. 3:**
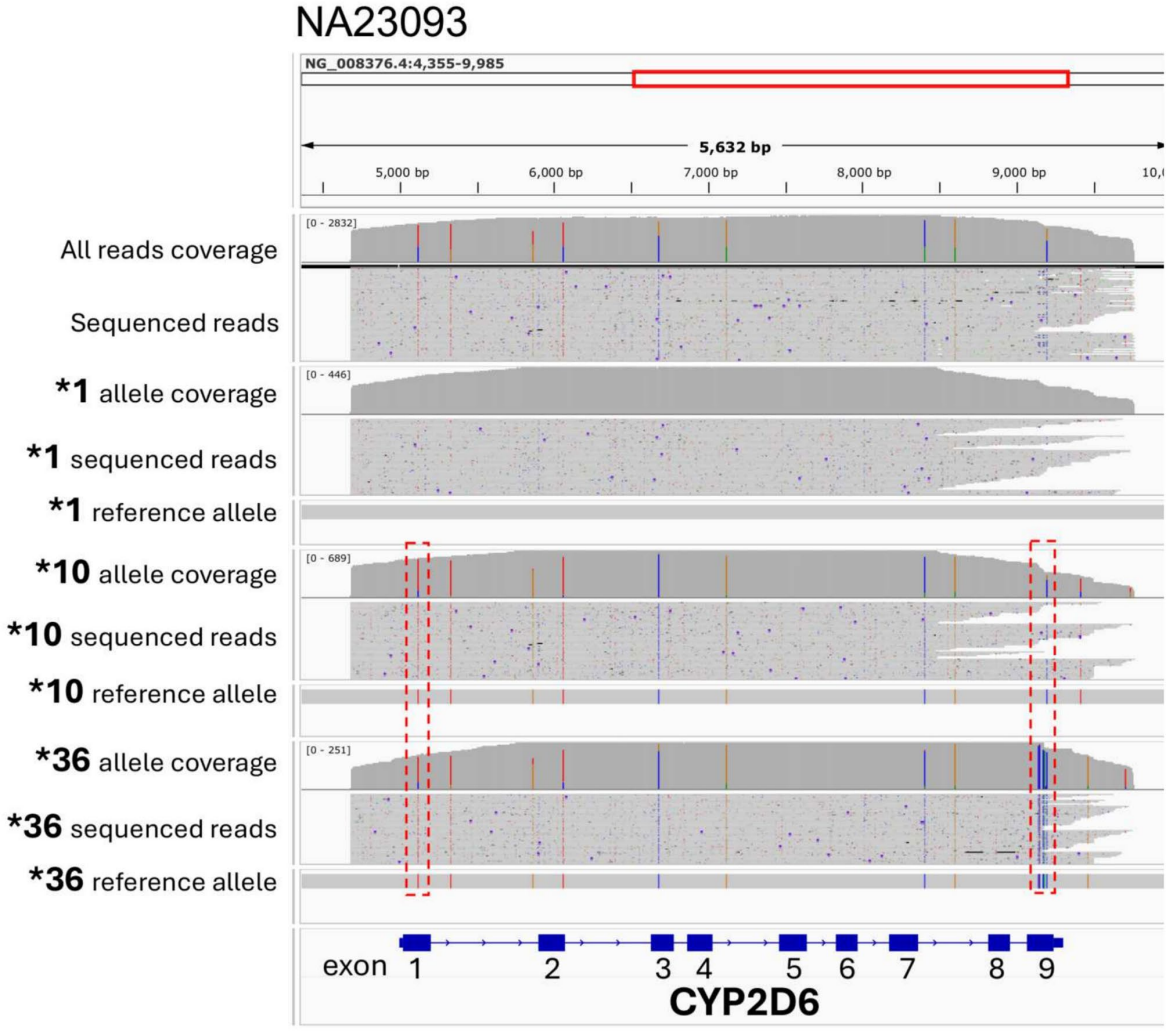
Integrative Genomics Viewer (IGV) plot validating *1, *10, and *36 allele in sample NA23093. The IGV plot displays all sequenced reads from NA23093, revealing a mix of three alleles: *1, *10, and *36. Reads corresponding to each allele are plotted separately, along with reference alleles, to confirm the presence of key variants. Red boxes highlight critical SNPs in exons 1 and 9 that distinguish *10 and *36 alleles. This visualization demonstrates the accurate identification and separation of alleles within the sample.

We compared the results of 3 tools, i.e., Stargazer^20^, Aldy^21^, and PyPGx^22^, for analyzing *CYP2D6* in the 12 samples. The tool with the highest accuracy was Stargazer which had F1 scores closest to 1, regardless of whether rapid library preparation (F1 = 0.92) or ligation library preparation (F1 = 0.83) was used. PyPGx (F1 = 0.50 for Rapid and 0.42 for Ligation) had the second highest accuracy, and Aldy had lowest accuracy (F1 = 0.33 for both Rapid and Ligation). When Stargazer was combined with Rapid library preparation, the results revealed that sample NA23093 had incorrect interpretation. Previous genotyping of sample NA23093 produced variations with different methods, indicating that this is a challenging sample, and resulted in *1/*10 or *1/*36^27^. In this case, we used IGV visualization tool to validate our results for sample NA23093 and identified *1, *10, and *36 (Fig. 3); however, manual verification is required. In this section, we summarize that the best results were obtained by combining our selected polymerase, PCR conditions, Rapid library preparation, and Stargazer. This combination would then be applied to 12 clinical samples, which have lower-quality DNA.

After amplifying the 12 clinical samples with PCR, we observed that the PCR amplicon sizes matched what we expected (5.1 kb) (Supplementary Fig. 2), confirming that the PCR conditions optimized are applicable to clinical samples. Then, only the Rapid library preparation method for nanopore sequencing is applied to sequence 12 clinical DNA samples. Interestingly, sample MS-172 did not show amplicon in *CYP2D6* gene copies but instead exhibited a faint band at 3.5 kb (Supplementary Fig. 2).

Again, we compared the accuracy of the 3 bioinformatics tools on the clinical samples (Table 2). As in 12 GeT-RM DNA standard studies, Stargazer emerged as the most accurate, achieving the highest F1 score (0.78), followed by PyPGx (F1 = 0.43), and Aldy had lowest accuracy (F1 = 0.35) (Table 2). Discrepancies between known genotypes and those produced by Stargazer were noted particularly in samples MS-158 and MS-172.

**Table 2:** Genotyping results and F1 score from three bioinformatics tools for twelve clinical cohorts using Rapid library preparation methods.

Get-RM ID	Diplotype (real-time PCR)	Aldy	PyPGx	Stargazer
MS-030	*1/*10	*1/*10	*2/*10	*1/*10
MS-043	*1/*10	*1/*10	*2/*10	*1/*10
MS-062	*1/*10	*10/*10	*10/*10	*10/*10
MS-113	*4/*10	*1/*1	*4/*10	*4/*10
MS-115	*1/*4	*1/*4	*2/*4	*1/*4
MS-116	*1/*4	*4M/*10	*2/*4	*1/*4
MS-143	*1/*2	*1/*2	*1/*2	*1/*2
MS-158	*2/*10	*34/*119	*2/*69	*10/*41
MS-159	*10/*10	*1/*1	*10/*10	*10/*10
MS-161	*10/*10	*1/*1	*10/*10	*10/*10
MS-169	*10/*10	*1/*1	*10/*10	*10/*10
MS-172	N*1/*2	NA	NA	NA
F1 score		0.35	0.43	0.78

Bold highlighting indicates a genotype consistency.

Italics highlighting signifies a genotype discrepancy; however, the updated result is accurate.

### Discrepancies between previously identified genotype and nanopore sequencing results

**Fig. 4:**
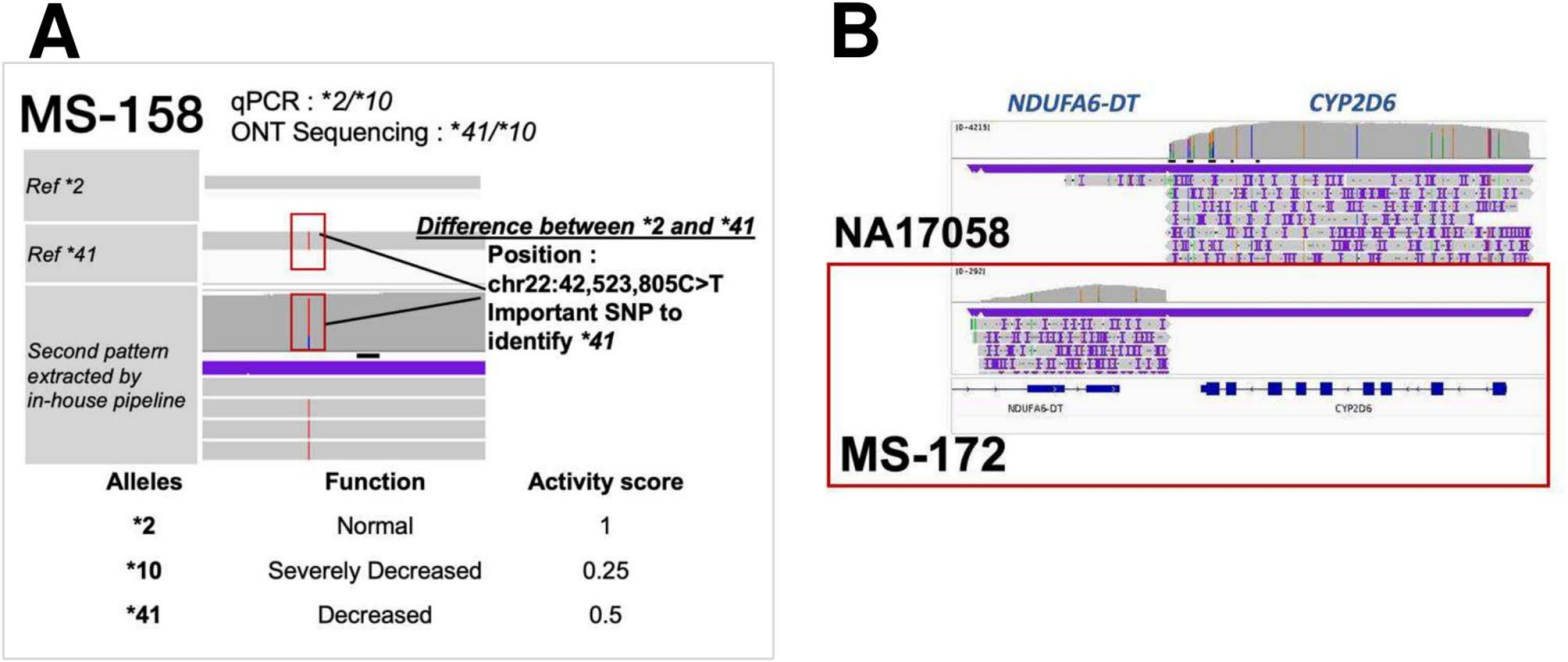
Nanopore sequencing of MS-158 and MS-172. (**A**) For MS-158, the analysis revealed a SNP indicative of the *41 allele pattern. (**B**) Nanopore sequencing of sample MS-172 did not detect *CYP2D6*.

To observe the discrepancies with samples MS-158 and MS-172, we plotted the sequencing data on IGV for detailed examination (Fig. 4A). The sequencing data showed that MS-158 contains a key SNP that links to *41 more than *2; therefore, this sample should be *10/*41, rather than *2/*10. More importantly, this allele could change gene function from “normal metabolite” (*2/*10; score 1.25) to “intermediate metabolite” (*10/*41; score 0.75) (Fig. 4A). For MA-172, the mapping result matched the PCR result, suggesting that the entire *CYP2D6* gene is deleted (Fig. 4B).

### Structural variation detection

Although Stargazer presented the most favorable initial results, our amplicon sequencing analysis revealed that it failed to detect certain structural variants, including duplications and various types of *CYP2D6-D7* hybrids. Specifically, for duplications, we depended on PCR amplification data and analyzed the amplicon ratios. For instance, in the case of the standard DNA sample NA02016, we observed two distinct bands (see Fig. 2). The smaller band, measuring 3.5 Kb, was located downstream of the *CYP2D6* gene (shown in Kanokpich Puaprasert et al.^19^). Subsequent quantification of the amplicon ratios revealed a predominance of the *2 allele over the *17 allele in sample NA02016.

**Fig. 5:**
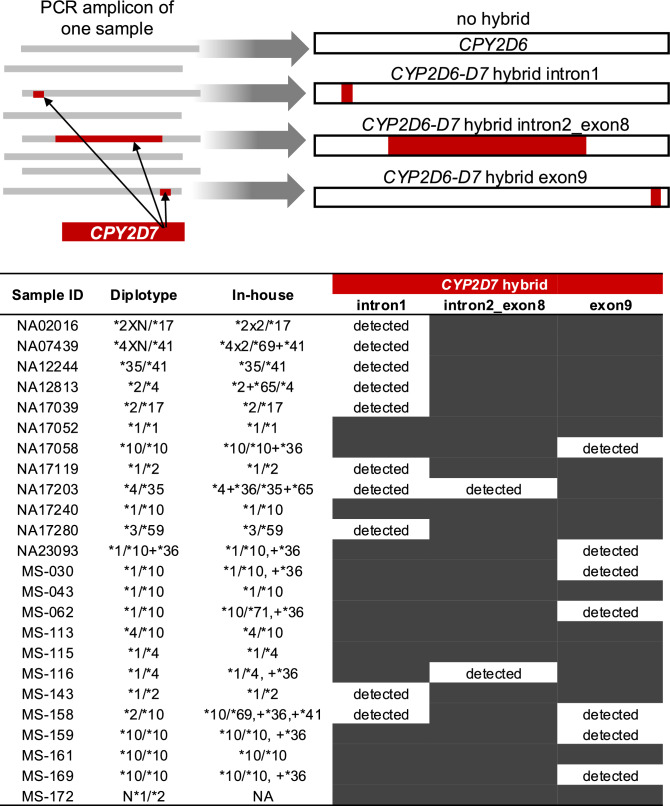
Detection of *CYP2D6-D7* hybrid structures in standard DNA samples. Upper Section: a schematic representation of the PCR amplicon for one sample, illustrating no hybrid, and various hybrid formations with *CYP2D7* including *CYP2D6-D7* hybrid intron1, *CYP2D6-D7* hybrid intron2_exon8, and *CYP2D6-D7* hybrid exon9. These hybrids are marked in red. Lower Section: a table summarizing the detection of specific hybrid formations. Each sample is listed along with a comparison between the known diplotype from GeT-RM or real-time PCR and the in-house genotype workflow. The table also indicates the presence of hybrids in intron1, intron2_exon8, and exon9 of the *CYP2D6-D7* genes.

In terms of *CYP2D6-D7* hybrids, there are two DNA standard samples that previously report of hybrid structure^28^ and we used in this study, i.e., NA17058 and NA17203. In our work, three star allele calling tools did not report the hybrid results. We then plotted the IGV (Supplementary Fig. 3) and found hybrids as previous report^28^. Each of these hybrid types incorporates segments of *CYP2D7* gene. Then we asked whether how many samples carry hybrids. Using IGV plot to observe the standard samples, we found many hybrid cases. We, then, reanalyzed 24 samples, comprising 12 DNA standard samples and 12 clinical samples, by classifying the sequencing data into four categories and using key SNPs by (1) sequence with no hybrid (no_hybrid), (2) sequence that can detect *CYP2D7* hybrid in intron1 (hybrid_intron1), (3) sequence that can detect *CYP2D7* hybrid in intron2 and exon8 (hybrid_intron2_exon8), (4) sequence that can detect *CYP2D7* hybrid in exon9 (hybrid_exon9) as shown in Fig. 5.

Our findings indicate that 17 of the 24 samples contained *CYP2D6-D7* hybrids, with various types of hybrids present in both the DNA standard and clinical sample sets. Given these findings, we perform in-house analysis by starting from separate the sequences into no_hybrid, hybrid_intron1, hybrid_intron2_exon8, and hybrid_exon9 categories, followed by haplotype phasing, consensus calling, and star allele typing, as detailed in the Methods section.

This refined analytical approach was further applied in Phase 2, which encompasses 90 cohorts, enabling a more detailed exploration of the genetic interactions and variations within this larger dataset.

### Analysis of *CYP2D6* genotypes, activity scores, and predicted phenotypes in 90 cohorts using stargazer and in-house methods

**Fig. 6:**
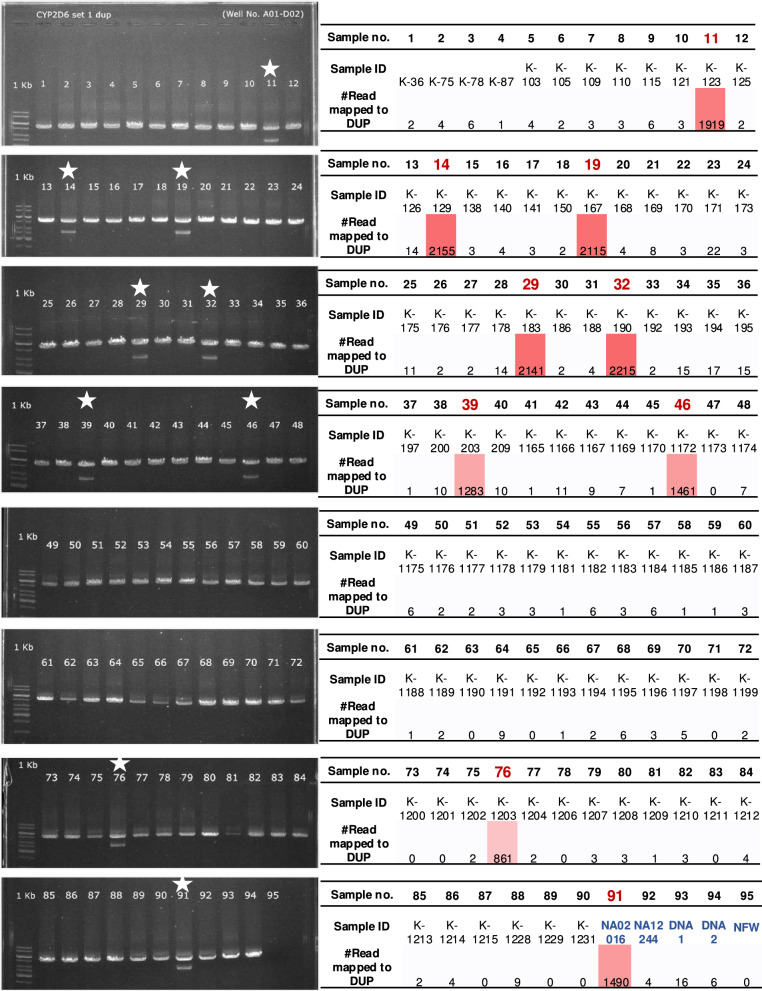
Agarose gel electrophoresis analysis and corresponding read mapping data for the *CYP2D6* gene and *CYP2D6* gene duplication. The left panel displays gel electrophoresis results for *CYP2D6* across 95 samples; 1 band indicates the presence of *CYP2D6*, and double bands indicate the presence of a gene duplication. Notable samples are marked with stars. The right panel is a tabular summary of the sample numbers (1 − 95), their corresponding IDs (K-36 to nuclease-free water [NFW]), and the number of reads mapped to the *CYP2D6* gene duplication (DUP). Red boxes highlight samples with significant read numbers mapped to the gene duplication, suggesting a positive result for *CYP2D6* duplication. Samples with high read counts also are outlined in red. The original gel images were shown in Supplementary Fig. 4A–H.

To obtain star allele on 90 DNA samples from a cohort of subjects from Ubon Ratchathani, a province in the Greater Mekong Subregion in Thailand, the Phase 1’s method was applied in this cohorts. PCR results revealed amplified *CYP2D6* genes in all samples from the Ubon Ratchathani cohort (Fig. 6). In addition to 90 Ubon Ratchathani cohorts, we also added 5 control samples into this phase, i.e., sample 91–95. The controls included positive control samples (91–94) and a negative control sample or nuclease-free water (95) to demonstrate the absence of DNA contamination. Sample 91 exhibited a *CYP2D6* duplication, and samples 92, 93, and 94 did not show any duplication. Reads obtained from the nanopore were mapped onto the human genome, revealing that a significant number of reads were mapped to the end of the *CYP2D6* gene in samples that had more than one copy of *CYP2D6*. The results obtained in this section could be used as a marker to indicate the pattern of the *CYP2D6* gene in terms of gene duplication.

The star allele, genotype, activity score, and predicted phenotype of 90 cohorts based-on in-house analysis and Stargazer are shown in the Table 3. The in-house analysis generally aligned with the Stargazer predictions in terms of activity scores and predicted phenotypes. Most discrepancies were observed in samples containing complex alleles, reflecting the sensitivity of the in-house method to specific allelic interactions that Stargazer might not fully account for. The majority of the samples exhibited *10/*10 alleles, both with and without the +*36 modifier, showing an activity score of 0.5 and an intermediate metabolizer (IM) phenotype. This was consistently observed in both the in-house and Stargazer analyses. Regarding the predicted phenotype, there are discrepancies in 11 out of 90 cohorts (12%) between the in-house and Stargazer analyses. Samples such as CYPTM006, which harbors the *10/*144, +*36 allele, and CYPTM008 and CYPTM009 with *10/*69, +*36 +*41 +*39 alleles, displayed significant differences in activity scores between the two analysis methods. For instance, CYPTM006 showed a lower activity score of 0.25 (IM) in the in-house analysis compared to 1.25 (NM) in Stargazer, indicating a potential overestimation of metabolic activity by Stargazer in complex genotypes. For alleles without complex modifiers, such as *1/*10 and *2/*2, both methods showed good consistency in predicting activity scores and phenotypes, reinforcing the reliability of genotyping tools in straightforward allelic backgrounds. In conclusion, the comparison between in-house and Stargazer genotyping results highlights both the challenges and strengths of current predictive methods in pharmacogenomics.

**Table 3:** Genotype, star allele, activity score, and predicted phenotype of 90 cohorts and 4 control samples based-on in-house analysis and Stargazer.

Sample ID	In-house analysis	Activity score	Predicted phenotype	Stargazer	Activity score	Predicted phenotype
CYPTM001	*10/*10, + *36	0.5	IM	*10/*10	0.5	IM
CYPTM002	*1/*10, + *36	1.25	NM	*1/*10	1.25	NM
CYPTM003	*1/*10, + *36	1.25	NM	*1/*10	1.25	NM
CYPTM004	*1/*10, + *36	1.25	NM	*1/*10	1.25	NM
CYPTM005	*10/*10, + *36	0.5	IM	*10/*10	0.5	IM
*CYPTM006*	*10/*144, + *36	*0.25*	***IM***	*1/*10	*1.25*	***NM***
CYPTM007	*10/*10, + *36	0.5	IM	*10/*10	0.5	IM
*CYPTM008*	*10/*69, + *36 + *41 + *39	*1.5*	***NM***	*10/*41	*0.5*	***IM***
*CYPTM009*	*10/*69, + *36 + *41 + *39	*1.5*	***NM***	*10/*41	*0.5*	***IM***
CYPTM010	*10/*10, + *36	0.5	IM	*10/*10	0.5	IM
*CYPTM011*	*1/*2 × *2*	*3*	***UM***	*1/*2	*2*	***NM***
CYPTM012	*10/*10, + *36 + *39	1.5	NM	*10/*39	1.25	NM
CYPTM013	*1/*144	1	IM	*1/*144	1	IM
*CYPTM014*	*1/*2 × *2*	*3*	***UM***	*1/*2	*2*	***NM***
CYPTM015	*10/*10, + *36	0.5	IM	*10/*10	0.5	IM
CYPTM016	*10/*10	0.5	IM	*10/*10	0.5	IM
CYPTM017	*2/*2	2	NM	*2/*2	2	NM
*CYPTM018*	*10/*71, + *36	*0.25*	***IM***	*1/*10	*1.25*	***NM***
CYPTM019	*2 + *65/*10 + *36 + *39	2.25	NM	*2/*10	1.25	NM
CYPTM020	*1/*10	1.25	NM	*1/*10	1.25	NM
CYPTM021	*10/*10, + *36	0.5	IM	*10/*10	0.5	IM
CYPTM022	*1/*10	1.25	NM	*1/*10	1.25	NM
CYPTM023	*1/*10	1.25	NM	*1/*10	1.25	NM
CYPTM024	*10/*10, + *36	0.5	IM	*10/*10	0.5	IM
CYPTM025	*1/*10	1.25	NM	*1/*10	1.25	NM
CYPTM026	*10/*10, + *36	0.5	IM	*10/*10	0.5	IM
CYPTM027	*10/*10, + *36	0.5	IM	*10/*10	0.5	IM
CYPTM028	*10/*10	0.5	IM	*10/*10	0.5	IM
CYPTM029	*1/*10, + *36	1.25	NM	*1/*10	1.25	NM
CYPTM030	*1/*10, + *36	1.25	NM	*1/*10	1.25	NM
CYPTM031	*1/*10, + *36	1.25	NM	*1/*10	1.25	NM
*CYPTM032*	*1/*2 × *2*	*3*	***UM***	*1/*2	*2*	***NM***
CYPTM033	*1/*1	2	NM	*1/*1	2	NM
*CYPTM034*	*10/*69, + *39 + *41	*1.5*	***NM***	*10/*41	*0.5*	***IM***
CYPTM035	*10/*10	0.5	IM	*10/*10	0.5	IM
CYPTM036	*1/*10	1.25	NM	*1/*10	1.25	NM
CYPTM037	*10/*10, + *36	0.5	IM	*10/*10	0.5	IM
CYPTM038	*1/*1	2	NM	*1/*1	2	NM
CYPTM039	*2/*2, + *41	2.25	NM	*2/*41	1.25	NM
CYPTM040	*1/*10	1.25	NM	*1/*10	1.25	NM
CYPTM041	*10/*10, + *36	0.5	IM	*10/*10	0.5	IM
CYPTM042	*1/*10	1.25	NM	*1/*10	1.25	NM
CYPTM043	*1/*41	1.25	NM	*1/*41	1.25	NM
CYPTM044	*10/*10, + *36	0.5	IM	*10/*10	0.5	IM
CYPTM045	*1/*10, + *36	1.25	NM	*1/*10	1.25	NM
CYPTM046	*4/*10, + *36	0.25	IM	*4/*10	0.25	IM
CYPTM047	*10/*10, + *36	0.5	IM	*10/*10	0.5	IM
*CYPTM048*	*10/*69, + *36 + *41 + *39	*1.5*	***NM***	*10/*41	*0.5*	***IM***
CYPTM049	*10/*10, + *36	0.5	IM	*10/*10	0.5	IM
CYPTM050	*1/*10	1.25	NM	*1/*10	1.25	NM
CYPTM051	*10/*10, + *36	0.5	IM	*10/*10	0.5	IM
CYPTM052	*1/*10, + *36	1.25	NM	*1/*10	1.25	NM
CYPTM053	*10/*10, + *36	0.5	IM	*10/*10	0.5	IM
*CYPTM054*	*10/*69, + *36 + *41 + *39	*1.5*	***NM***	*10/*41	*0.5*	***IM***
CYPTM055	*1/*10, + *36	1.25	NM	*1/*10	1.25	NM
CYPTM056	*1/*2	2	NM	*1/*2	2	NM
CYPTM057	*1/*10, + *36	1.25	NM	*1/*10	1.25	NM
CYPTM058	*1/*10	1.25	NM	*1/*10	1.25	NM
CYPTM059	*10/*69, + *36 + *41 + *39	1.5	NM	*10/*41	0.5	NM
CYPTM060	*1/*10, + *36	1.25	NM	*1/*10	1.25	NM
CYPTM061	*10/*39	1.25	NM	*10/*39	1.25	NM
CYPTM062	*10/*10, + *36	0.5	IM	*10/*10	0.5	IM
CYPTM063	*10/*10, + *36	0.5	IM	*10/*10	0.5	IM
CYPTM064	*4/*69, + *41	0.25	IM	*4/*41	0.25	IM
CYPTM065	*10/*10, + *36	0.5	IM	*10/*10	0.5	IM
CYPTM066	*4/*10	0.25	IM	*4/*10	0.25	IM
CYPTM067	*10/*10, + *36	0.5	IM	*10/*10	0.5	IM
*CYPTM068*	*1/*10, + *36	*1.25*	***NM***	*10/*10	*0.5*	***IM***
CYPTM069	*10/*10	0.5	IM	*10/*10	0.5	IM
CYPTM070	*10/*10, + *36	0.5	IM	*10/*10	0.5	IM
CYPTM071	*10/*10, + *36	0.5	IM	*10/*10	0.5	IM
CYPTM072	*1/*10, + *36	1.25	NM	*1/*10	1.25	NM
CYPTM073	*1/*10, + *36	1.25	NM	*1/*10	1.25	NM
CYPTM074	*10/*69, + *36 + *41 + *39	1.5	NM	*10/*41	0.5	NM
CYPTM075	*1/*10	1.25	NM	*1/*10	1.25	NM
CYPTM076	*2/*10, + *65 + *39	2.25	NM	*2/*10	1.25	NM
CYPTM077	*41/*41	0.5	IM	*41/*41	0.5	IM
CYPTM078	*1/*10, + *36	1.25	NM	*1/*10	1.25	NM
**CYPTM079**	*10/novel	**0.25**	**IM**	*1/*10	**1.25**	**NM**
CYPTM080	*1/*1	2	NM	*1/*1	2	NM
CYPTM081	*10/*10	0.5	IM	*10/*10	0.5	IM
CYPTM082	*10/*10, + D6-D7 × 2	0.5	IM	*10/*10	0.5	IM
CYPTM083	*10/*10, + *36	0.5	IM	*10/*10	0.5	IM
CYPTM084	*10/*10, + *36	0.5	IM	*10/*10	0.5	IM
CYPTM085	*10/*10, + *36	0.5	IM	*10/*10	0.5	IM
CYPTM086	*1/*1	2	NM	*1/*1	2	NM
CYPTM087	*10/*10, + *36	0.5	IM	*10/*10	0.5	IM
CYPTM088	*10/*10, + *36	0.5	IM	*10/*10	0.5	IM
CYPTM089	*2/*10, + *65 + *39	2.25	NM	*2/*10	1.25	NM
CYPTM090	*2/*10, + *65 + *39	2.25	NM	*2/*10	1.25	NM
*CYPTM091*	*2/*2, + *17	*2.5*	***UM***	*2/*17	*1.5*	***NM***
CYPTM092	*35,*41,*2	2.25	NM	*35/*41	1.25	NM
CYPTM093	*1/*10	1.25	NM	*1/*10	1.25	NM
CYPTM094	*10/*10	0.5	IM	*10/*10	0.5	IM

UM: Ultrarapid metabolizer (Increased enzyme activity); NM: Normal metabolizer (Fully functional enzyme activity); IM: Intermediate metabolizer (Decreased enzyme activity).

Text in bold indicates samples containing a novel allele.

Areas highlighted in italics denote discrepancies between in-house analysis and Stargazer results.

### Frequency and activity of *CYP2D6* alleles in the 90 cohorts.

**Fig. 7:**
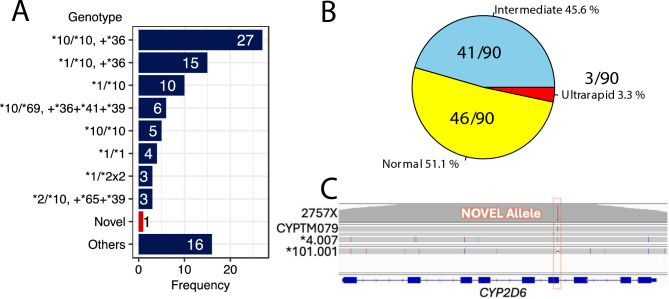
*CYP2D6* genotype and activity frequency in the dataset of 90 samples. (**A**) Genotype frequency. (**B**) Distribution of *CYP2D6* phenotypes. (**C**) Potential novel allele found in this study.

The distribution of genotypes among the study sample is summarized in Fig. 7A. The most frequent genotype observed was *10/*10, + *36, present in 27 individuals, accounting for approximately 30% of the cohort. This was followed by *1/*10, + *36 with 15 individuals and *10/*69, + *36 + *41 + *39 with 6 individuals. Notably, a novel *CYP2D6* allele was identified in one individual, highlighting the genetic variability within this population. Genotypes classified as 'Others,' which include various combinations not specifically charted, were observed in 16 individuals.

The distribution of CYP2D6 phenotypes among the 90 samples is illustrated in the Fig. 7B. Phenotypic classification based on metabolic activity shows that 51.1% of the individuals were categorized as normal metabolizers, the most common phenotype. Intermediate metabolizers constituted 45.6% of the population, while ultrarapid metabolizers were identified in 3.3%, underscoring the potential for varied drug metabolism and efficacy within this group. Figure 7C, the locus-specific depiction of the novel allele in relation to known *CYP2D6* sequences. This novel allele, positioned uniquely on the *CYP2D6* gene map, suggests alterations that may impact enzyme function, although further studies are needed to elucidate its specific effects on drug metabolism. These results underscore the genetic diversity in *CYP2D6* alleles within the Greater Mekong Sub-region population and indicate significant implications for personalized medicine approaches in drug therapy.

### Cost analysis of Rapid library kits across various sample sizes

**Fig. 8:**
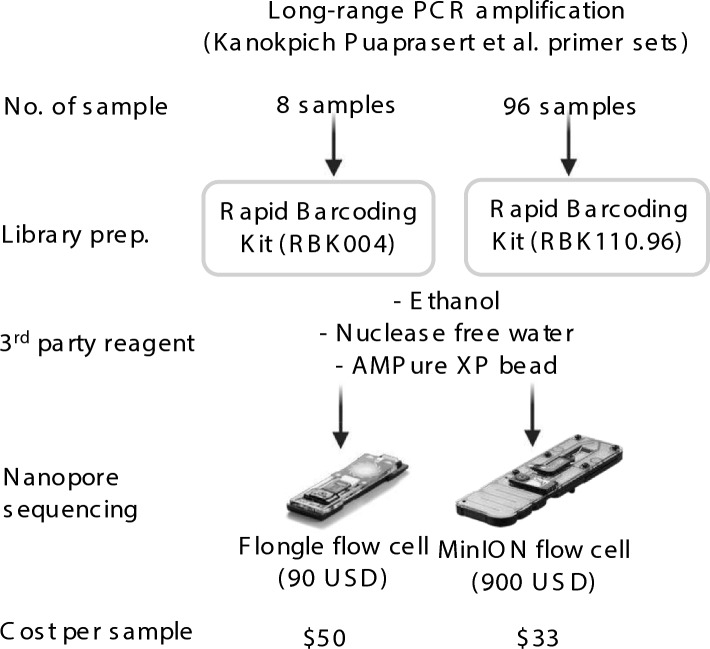
Cost for nanopore sequencing using long-range PCR amplification approach.

Figure 8 illustrates a comparative analysis of the costs involved in nanopore sequencing using long-range PCR amplification and Rapid library preparation for two different sample sizes, 8 samples and 96 samples, utilizing different Rapid Barcoding Kits (RBK004 and RBK110.96, respectively). For the sequencing step, two types of flow cells are used: the Flongle flow cell and the MinION flow cell, each with distinct cost implications. The Flongle flow cell, costing 90 USD, is employed for the smaller sample size (8 samples), resulting in a cost of 50 USD per sample. In contrast, the MinION flow cell, also priced at 900 USD, is used for the larger sample size (96 samples), leading to a significantly reduced cost of 33 USD per sample. This cost analysis highlights the scalability and cost-effectiveness of using different flow cells based on the number of samples processed. The MinION flow cell proves to be more economical for larger sample sizes due to the reduced cost per sample, whereas the Flongle flow cell is more suitable for smaller-scale studies. This comparison underscores the importance of choosing the appropriate sequencing setup based on the specific needs and scale of the study, particularly in terms of budget constraints and sample throughput requirements.

## Discussion

To prevent relapses of *P. vivax* malaria, primaquine is critical, and its ability to achieve a radical cure is largely attributed to metabolism mediated by the CYP2D6 enzyme^14,29^. Tafenoquine, a long-acting analogue of primaquine, has been developed to provide a radical cure for P. vivax malaria. However, there are conflicting findings regarding the impact of CYP2D6 metabolism on the therapeutic efficacy of tafenoquine. For instance, one clinical study indicated that variations in CYP2D6 activity did not influence the effectiveness of tafenoquine in patients infected with *P. vivax*. Conversely, pharmacokinetic studies in CYP2D knockout mice revealed significant differences in tafenoquine metabolism compared to wild-type mice, suggesting that CYP2D6 might indeed affect the drug’s pharmacological profile^30–32^. Further investigations are necessary to clarify the role of *CYP2D6* mutations in the biological activity of tafenoquine.

The CYP2D6 gene is highly polymorphic, with hundreds of variant alleles identified, potentially influencing the metabolism of a wide range of clinically used medications. Previous studies that have used nanopore sequencing for *CYP2D6* genotyping relied on ligation library preparation^33,34^, which requires multiple enzymes and can complicate logistics, especially in field conditions of low-to-middle − income countries. There is a need for simple, cost-effective methods to prepare DNA libraries, followed by nanopore sequencing technology to facilitate point-of-care diagnostics and immediate clinical decision-making.

Although this study is not the first to apply nanopore sequencing to the *CYP2D6* gene, it is the first to utilize the Rapid library preparation kit. This kit significantly simplifies the protocol and minimizes the need for multiple enzymes from third-party suppliers. In Southeast Asian countries such as Thailand and Indonesia, the procurement of reagents and enzymes is complicated by additional taxes and margins, as well as import times that can extend to several weeks up to several months. The adoption of the Rapid Barcoding Kit mitigates these logistical challenges by streamlining the workflow and reducing dependency on external vendors, thereby improving the feasibility and efficiency of sequencing efforts in these regions.

In this study, we utilized the R9.4.1 version of the flow cell and the RBK110.96 Rapid Barcoding Kit, which are not the latest versions available. The newer R10.4.1 version offers improved accuracy, particularly in homopolymer sequencing, while maintaining the same cost. Future studies could benefit from using the updated R10.4.1 version to enhance sequencing accuracy without incurring additional expenses.

Compared to the study by Wankaew et al., which used short-read whole genome sequencing on 171 Thai individuals, our results align in terms of the most prevalent allele, specifically CYP2D6*10 + 36 and *10, which are associated with the intermediate metabolizer phenotype^35^. Our research further enriches the dataset with information on complex structural variants involving *CYP2D6* and its pseudogene, *CYP2D7*. This aligns with findings by Yaowaluck Hongkaew et al.^36^, which demonstrated that the *CYP2D6* gene in the Thai population features hybrid gene structures, underscoring the necessity for systematic characterization of the *CYP2D6* locus across diverse populations, including Thai.

The advantages of sequencing over real-time PCR, as demonstrated in this study, include the ability to detect novel alleles and gain detailed insights into structural variations (SVs) through long-read sequencing. Moreover, the refinement of star alleles, such as *41, has the potential to alter the phenotype of the sample, offering a more accurate and comprehensive understanding of genetic variation. Refined diplotypes did not result in predicted phenotype change in most cases, however, nanopore-based calls predict an indeterminate rather than normal metabolizer call for *CYP2D6* in the control sample.

We have detailed an in-house data analysis workflow in Supplementary Fig. 1, in which nanopore reads are sorted into subclasses of structural variants (SVs) and, where feasible, phased before being compiled into precise haplotypes. This methodology, used to analyze 90 samples from the Greater Mekong Sub-region in Thailand, effectively profiles common alleles and identifies novel ones. Demonstrating significant potential, this approach could be expanded to accommodate larger sample sizes in subsequent research phases. Furthermore, it paves the way for the development of specialized software tailored for the nanopore rapid sequencing kit.

The integration of our experimental protocol with bioinformatics analysis provides a streamlined approach that can deliver same-day results when paired with star allele calling using Stargazer. In cases where structural variations are considered, our in-house bioinformatics workflow provides a robust foundation for analysis and will be developed into user-friendly software in future work. As part of this effort, ongoing developments will be focused on creating a streamlined bioinformatics pipeline using Nextflow, a modern workflow management system tailored for bioinformatics applications. Nextflow enables the creation of scalable, reproducible, and portable workflows by leveraging containerized environments such as Docker and Singularity, ensuring seamless compatibility across diverse computing platforms, including local machines, high-performance clusters (HPCs), and cloud systems. By integrating intuitive features and graphical user interfaces, the pipeline will simplify complex analyses, making data processing more accessible to field personnel with limited bioinformatics expertise. These advancements aim to enhance the scalability and usability of the workflow, providing deeper insights into metabolizer phenotypes and potential mechanisms underlying malaria relapse.

To effectively eliminate *P. vivax* malaria, there needs to be a broader implementation of primaquine treatment at appropriate dosages. The optimal dosage may vary depending on the prevalent CYP2D6 mutations within different populations. Therefore, further research is essential to investigate this relationship across diverse groups to ensure appropriate dosing strategies. The development of the described genotyping techniques could play a crucial role in facilitating this research.

## Conclusion

This study presents an alternative method for in-field delivery of ultra-high-resolution *CYP2D6* genotype data, and phenotype prediction offering a unique opportunity for point-of-care diagnostics. The combination of a Rapid kit and nanopore sequencing significantly enhances the ability to quickly understand the genetic makeup of the *CYP2D6* gene. The findings propose possibilities that genetic variation could be incorporated to assist policymakers in making (near) real-time decisions based on accurate information, thereby potentially enhancing the effectiveness of *P. vivax* elimination.

## Supplementary Information

Supplementary Information.

## Data Availability

The datasets generated and analysed during the current study are available in the Sequence Read Archive (SRA) database under the BioProject ID PRJNA1190184.
