# Supplementary material for: Enhancing CYP2D6 genotyping with nanopore sequencing to address allele diversity P. vivax malaria elimination
Source: Sci Rep. 2025 Sep 30;15:33842. doi: 10.1038/s41598-025-05065-2 (PMC12484770; doi:10.1038/s41598-025-05065-2)
Supplement: Supplementary file 1 — Supplementary Information. [file 41598_2025_5065_MOESM1_ESM.pdf]

1 Purpose

1.1 To sequence human *CYP2D6* gene using MinION flow cell

2 Objective

2.1 To PCR amplify of *CYP2D6* gene, duplication, and deletion detection

2.2 To perform transposase-based sequencing using Rapid Barcoding Kit by Oxford Nanopore Technologies (ONT).

3 Materials and Consumables

3.1 200 ng DNA input

3.2 PCR amplification for *CYP2D6* gene, duplication, and deletion (modified from Puaprasert, et al., 2018)

3.2.1 5X PrimeSTAR GXL Buffer (R050A)

3.2.2 PrimeSTAR GXL DNA Polymerase (R050A)

3.2.3 dNTP Mixture (2.5 mM each)

3.2.4 primer DPKup_CYP2D6 (10 μM) (shown in Table 1)

3.2.5 primer DPKlow_CYP2D6 (10 μM)

3.2.6 primer F_2D6dupl (10 μM)

3.2.7 primer R_2D6dupl (10 μM)

3.2.8 primer 5′2D6*5 (10 μM)

3.2.9 primer 3′2D6*5 (10 μM)

3.3 Nuclease free water (B1500L, NEB)

3.4 Rapid Barcoding Kit: RBK114.24

3.5 R10.4.1 MinION flow cell

3.6 Nuclease-free water (B1500L, NEB)

3.7 Bovine Serum Albumin (BSA), Invitrogen™ UltraPure™ BSA 50 mg/ml, AM2616

3.8 Absolute Ethanol (200 proof), RNase-free; Fisher Cat. BP2818-500)

3.9 Freshly-prepared 80% ethanol in nuclease-free water (3 ml total)

3.10 Qubit™ dsDNA BR Assay Kit (Catalog number: Q32850)

3.11 1.5 mL microfuge tube (Eppendorf™ 022431021) and rack

3.12 Qubit™ Assay Tubes (Q32856) (in case user needs to check the concentration of the PCR amplicon concentration)

3.13 5 ml Eppendorf DNA LoBind tubes

3.14 PCR strip (Thermo Scientific™ ABgene™ EasyStrip™ AB2000)

3.15 Filter tip 10 μl, 20 μl, 200 μl, 1000 μl

3.16 Pipette set (1000, 200, 100, 10 μl)

3.17 Magnetic bead (Beckman Coulter: A63881) – store at 4°C (DO NOT FREEZE)

3.18 Biohazard bag
3.19 Gloves
3.20 Microfuge
3.21 Vortex mixer
3.22 Thermal cycler (PCR machine)
3.23 Qubit™ Fluorometer
3.24 Ice bucket with ice
3.25 Spin down centrifuge (for PCR strip and 1.5 ml tube)
3.26 Magnetic separation rack (0.2 μl and 1.5 ml) (NEB: S1506S)
3.27 Timer
3.28 MinION MK1B/ Mk1C/ GridION/ Flongle adaptor (for Flongle flow cell)
3.29 Computer PC/ Laptop (16 Gb RAM, 500 Gb space available)
3.30 Tapestation system/ gel electrophoresis

Table 1. Primer list.

ID	Primer 5'→3'	Expected amplicon size
2DPKup	GTTATCCCAGAAGGCTTTGCAGGCTTCA	5.1 Kb
2DPKlow	GCCGACTGAGCCCTGGGAGGTAGGTA	5.1 Kb
2D6dupl-F	CCTGGGAAGGCCCCATGGAAG	3.5 Kb
2D6dupl-R	CAGTTACGGCAGTGGTCAGCT	3.5 Kb
5′2D6*5	CACCAGGCACCTGTACTCCTC	3.5 Kb
3′2D6*5	CAGGCATGAGCTAAGGCACCCAGAC	3.5 Kb

Thidathip **Wongsurawat**
Thidathip.won@mahidol.edu
Mallika **Imwong**
mallika.imw@mahidol.ac.th

4 Procedure

4.1 PCR amplification of *CYP2D6* gene, duplication, and deletion

DNA concentration of PCR amplicon should be at least 50 ng/µl by Qubit assay**

1 DNA sample for 2 PCR reactions (200 ng DNA per reaction)

Tube 1

**Reagent**	**µl**
5X PrimeSTAR GXL Buffer	5
dNTP Mixture (2.5 mM each)	2
primer 2DPKup (10 µM)	1
primer 2DPKlow (10 µM)	1
primer 2D6dupl-F (10 µM)	0.5
primer2D6dupl-R (10 µM)	0.5
PrimeSTAR GXL DNA Polymerase	1
Nuclease free water + DNA	14
**Total**	**25**

Tube 2

**Reagent**	**µl**
5X PrimeSTAR GXL Buffer	5
dNTP Mixture (2.5 mM each)	2
primer 2DPKup (10 µM)	1
primer 2DPKlow (10 µM)	1
primer 5′2D6*5 (10 µM)	0.5
Primer 3′2D6*5 (10 µM)	0.5
PrimeSTAR GXL DNA Polymerase	1
Nuclease free water + DNA	14
**Total**	**25**

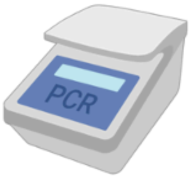

Temp.	Time	cycle
98°C	30 sec	1
98°C	10 sec	32
68°C	45 sec	
12°C	hold	∞

PCR Setup: Two reactions per samples would be performed. Totaling 200 ng for one PCR reaction. For each of the two PCR reactions, the following reagents are used in the specified volumes:

- ☐ 5X PrimeSTAR GXL Buffer: 5 µL
- ☐ dNTP Mixture (2.5 mM each): 2 µL
- ☐ Nuclease-Free Water + DNA: 14 µL

Tube 1:

- ☐ Primer 2DPKup (10 µM): 1 µL
- ☐ Primer 2DPKlow (10 µM): 1 µL
- ☐ Primer 2D6dupl-F (10 µM): 0.5 µL
- ☐ Primer 2D6dupl-R (10 µM): 0.5 µL

Tube 2:

- ☐ Primer 2DPKup (10 µM): 1 µL
- ☐ Primer 2DPKlow (10 µM): 1 µL
- ☐ Primer 5'2D6*5 (10 µM): 0.5 µL
- ☐ Primer 3'2D6*5 (10 µM): 0.5 µL

Each reaction mixture totals 24 µL

- ☐ Add PrimeSTAR GXL DNA Polymerase: 1 µL in to each tube

PCR Cycling Conditions:

- ☐ Initial Denaturation: 98°C for 30 seconds (1 cycle)
- ☐ Denaturation: 98°C for 10 seconds (32 cycles)
- ☐ Annealing/Extension: 68°C for 45 seconds (32 cycles)
- ☐ Final Hold: 12°C indefinitely

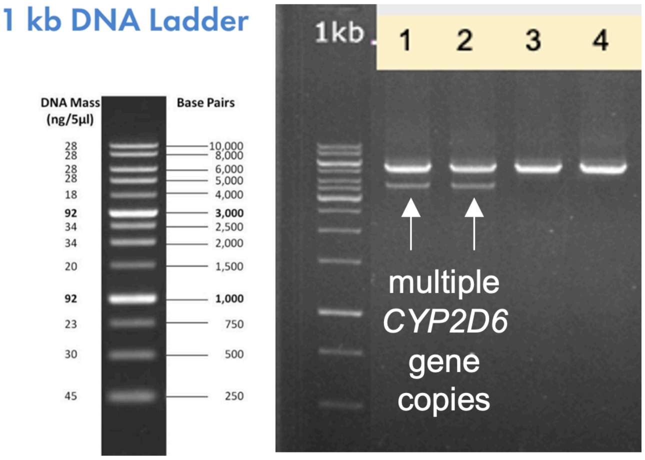

Expected Results Interpretation:
Lane 1-2 : Displays multiple bands (5.1 Kb and 3.5 Kb), suggesting the presence of multiple copies of the *CYP2D6* gene. These could be different alleles or duplications within the genome of this sample.
Lane 3-4: Shows a single distinct band, indicating a possible single copy of the *CYP2D6* gene or a specific single PCR product.

4.2 Library preparation and nanopore sequencing

- ☐ Starting Material: The protocol begins with an amplicon in "Tube 1." This tube contains an unspecified amount of amplicon.
- ☐ Transfer of Amplicon: From Tube 1, a specific volume of 3 µL of the amplicon is transferred to a new tube.
- ☐ Addition of Nuclease-Free Water: To the new tube containing the 3 µL amplicon, 7 µL of nuclease-free water is added.
- ☐ Resulting Mixture: The final mixture in the new tube now contains a total volume of 10 µL, consisting of the original amplicon diluted with nuclease-free water.
- ☐ Mix and spin down. The mixture will be used for library preparation step.

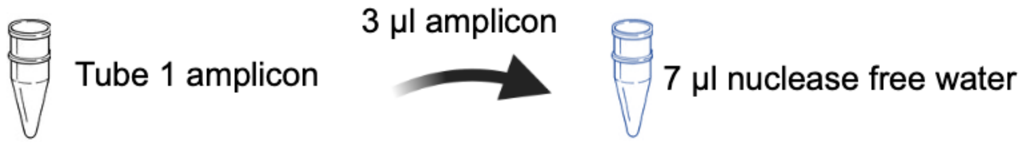

4.3 Library preparation and sequencing

Rapid Barcoding Kit 24 and 96 V14 (SQK-RBK114.24 or SQK-RBK114.96) protocol outlines the procedure for quick barcoding of input DNA. This SOP follows the original protocol Version: RBK_9176_v114_revM_27Nov2022. (https://community.nanoporetech.com/docs/prepare/library_prep_protocols/rapid-sequencing-gdna-barcoding-sqk-rbk114/v/rbk_9176_v114_revm_27nov2022/overview-of-the-protocol?devices=minion)

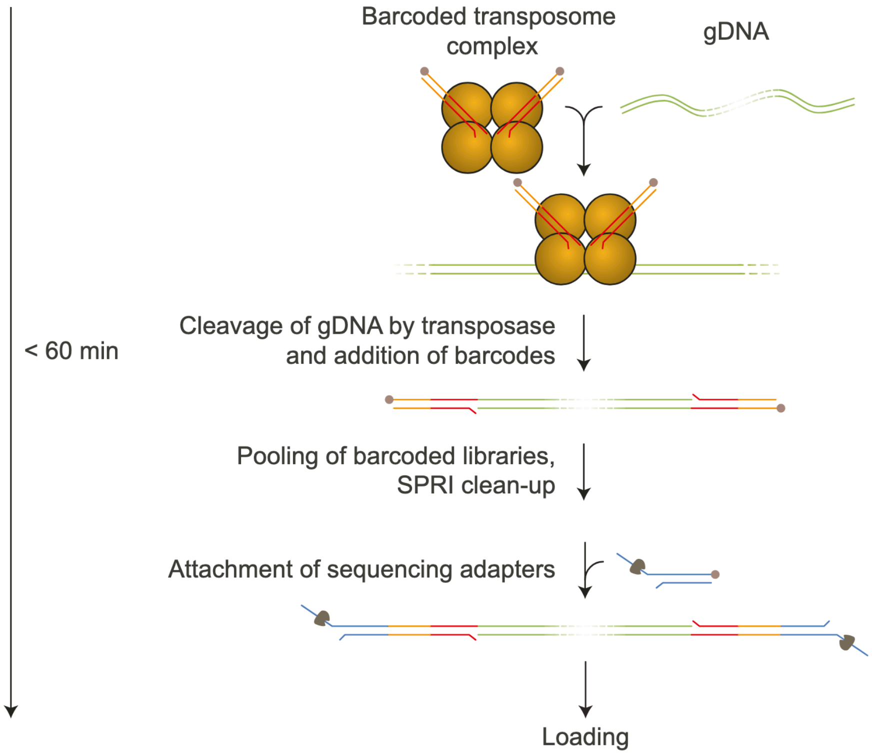

Library preparation: Rapid Barcoding Kit

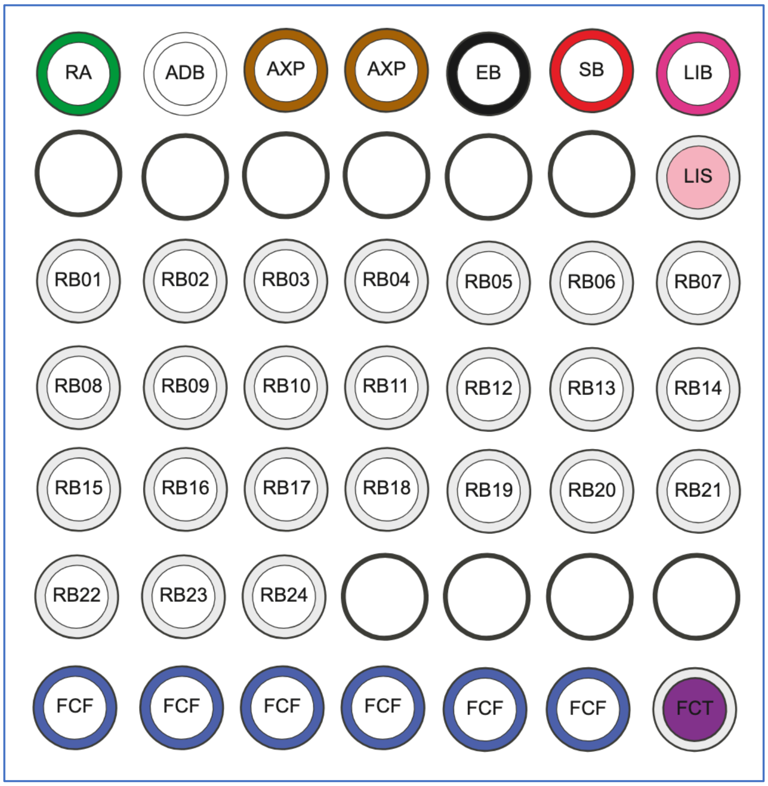

RA: Rapid Adapter
ADB: Adapter Buffer
LIB: Library Beads
LIS: Library Solution
EB: Elution Buffer
AXP: AMPure XP Beads
SB: Sequencing Buffer
FCF: Flow Cell Flush
FCT: Flow Cell Tether
RB01: Rapid Barcode 1
RB02: Rapid Barcode 2
RB03: Rapid Barcode 3
RB04: Rapid Barcode 4
RB05: Rapid Barcode 5
RB06: Rapid Barcode 6
RB07: Rapid Barcode 7
RB08: Rapid Barcode 8
RB09: Rapid Barcode 9
RB10: Rapid Barcode 10
RB11: Rapid Barcode 11
RB12: Rapid Barcode 12
RB13: Rapid Barcode 13
RB14: Rapid Barcode 14
RB15: Rapid Barcode 15
RB16: Rapid Barcode 16
RB17: Rapid Barcode 17
RB18: Rapid Barcode 18
RB19: Rapid Barcode 19
RB20: Rapid Barcode 20
RB21: Rapid Barcode 21
RB22: Rapid Barcode 22
RB23: Rapid Barcode 23
RB24: Rapid Barcode 24

Rapid Barcoding Kit (SQK-RBK114.24) contents

4.3.1 Barcoding and adaptor ligation

- ☐ 10 µl Template DNA (~200 ng from previous step)
- ☐ 1.5 µl Rapid Barcodes (RB01-24 or RB01-96, one for each sample)
- ☐ Incubate the tubes or plate at 30°C for 2 minutes and then at 80°C for 2 minutes. Briefly put the tubes or plate on ice to cool.
- ☐ Spin down the tubes or plate to collect the liquid at the bottom.
- ☐ Pool all barcoded samples in a clean 2 ml Eppendorf DNA LoBind tube, noting the total volume.
- ☐ Resuspend the AMPure XP Beads (AXP) by vortexing.
- ☐ Add an equal volume of resuspended AMPure XP Beads (AXP) to the entire pooled barcoded sample, and mix by flicking the tube.
- ☐ Incubate for 10 minutes at room temperature (RT).
- ☐ Prepare at least 2 ml of fresh 80% ethanol in Nuclease-free water.
- ☐ Bead clean up using Ampure beads (see below***)
- ☐ Remove the tube from the magnetic rack and resuspend the pellet in 15 µl Elution Buffer (EB) - For 24 barcodes: 15 µl; 96 barcodes: 60 µl
- ☐ Incubate for 10 minutes at RT.
- ☐ Pellet the beads on a magnet until the eluate is clear and colourless, for at least 1 minute.
- ☐ Transfer 11 µl of the sample into a clean 1.5 ml Eppendorf DNA LoBind tube.
- ☐ In a fresh 1.5 ml Eppendorf DNA LoBind tube, dilute the Rapid Adapter (RA) as follows and pipette mix:
- ☐ 1.5 µl Rapid Adapter (RA)
- ☐ 3.5 µl Adapter Buffer (ADB)
- ☐ Add 1 µl of the diluted Rapid Adapter (RA) to the barcoded DNA.
- ☐ Mix gently by flicking the tube, and spin down.
- ☐ Incubate the reaction for 5 minutes at RT

4.3.2 Priming and loading flow cell

Combine the following reagents in a fresh 1.5 ml Eppendorf DNA

- ☐ 1,170 µl Flow Cell Flush (FCF)
- ☐ 5 µl Bovine Serum Albumin (BSA)
- ☐ 30 µl Flow Cell Tether (FCT)
- ☐ 1,205 µl Final total volume in tube
- ☐ Slide the flow cell priming port cover clockwise to open the priming port.
- ☐ After opening the priming port, check for a small air bubble under the cover. Draw back a small volume to

- ☐ remove any bubbles:
- ☐ Set a P1000 pipette to 200 µl
- ☐ Insert the tip into the priming port
- ☐ Turn the wheel until the dial shows 220-230 µl, to draw back 20-30 µl, or until you can see a small volume
- ☐ of buffer entering the pipette tip
- ☐ Note: Visually check that there is continuous buffer from the priming port across the sensor array.
- ☐ Load 800 µl of the priming mix into the flow cell via the priming port, avoiding the introduction of air bubbles. Wait for 5 minutes. During this time, prepare the library for loading by following the steps below.
- ☐ Thoroughly mix the contents of the Library Beads (LIB) by pipetting.
- ☐ In a new 1.5 ml tube, prepare the library for loading as follows:
    - 37.5 µl Sequencing Buffer (SB)
    - 25.5 µl Library Beads (LIB) mixed immediately before use, or Library Solution (LIS), if using
    - 12 µl DNA library
- ☐ Complete the flow cell priming:
- ☐ Gently lift the SpotON sample port cover to make the SpotON sample port accessible.
- ☐ Load 200 µl of the priming mix into the flow cell priming port (not the SpotON sample port), avoiding the introduction of air bubbles.
- ☐ Mix the prepared library gently by pipetting up and down just prior to loading.
- ☐ Add 75 µl of the prepared library to the flow cell via the SpotON sample port in a dropwise fashion.
- ☐ Gently replace the SpotON sample port cover, making sure the bung enters the SpotON port and close the priming port.
- ☐ Gently lower the light shield onto the flow cell.
- ☐ Close the device lid and set up a sequencing run on MinKNOW.
- ☐ Run for 1-48 hours depends on number of the sample (30 mins per sample)
- ☐ Move files for downstream data analysis

**** Determining DNA concentration using Qubit**

- *Concentration:* Apply 1 µl of DNA. Use the Qubit™ to measure concentration (see ref. 8)
- *Integrity:* Apply 1-5 µl of DNA. Use the 0.8% Agarose gel to check the integrity (reference no. 4). Load 1 kb DNA ladder on the first lane and high molecular weight DNA ladder on the last

lane. After running the gel electrophoresis, high integrity of DNA will locate above 10 kb in the area of high molecular weight DNA ladder.

*** **Bead clean up using Ampure beads** (modified from reference no. 2)

- Allow magnetic bead reagent to come to room temperature prior to use.
- Mix the reagent well before use. It should appear homogenous and consistent in color (called mixture).
- Add 1 volume of the mixture to 1 volume of sample. For example, adding 80 µl bead to 80 µl DNA amplicons.
- Gently pipette up and down 10 times and gently flick the tube for 5 times then spin down (1 second).
- Incubate the mixture at room temperature for 5-10 minutes.
- Place the mixture onto magnetic separation rack for 3 minutes to separate beads from solution
- Prepare fresh 80% ethanol (800 µl ethanol and 200 µl NFW).
- Aspirate the cleared solution from the reaction tube and discard. This step must be performed while the reaction tube is situated on the magnetic separation rack. Do not disturb the area of magnetic beads.
- Dispense 200 µl of 80% ethanol to reaction tube and incubate for 1 minute at room temperature. Aspirate out the ethanol and discard. Repeat for a total of two washes.
- Remove the tube from magnetic separation rack and spin down the tube (1 second). Then, place the tube onto magnetic separation rack and use 10 µl pipette to aspirate out the remained ethanol and discard.
- Place the reaction tube on bench top to air-dry for 1 minute. Be sure to allow the dry
- Add 15 µl of elution buffer to the tube of the reaction and flicking 5 times or until no clump and perform spin down (1-2 second). And follow the protocol.

5 Safety

5.1 Waste solution must be stored in a right waste container / bag.

5.2 Be careful to minimize generation of aerosol by pipetting.

References

1. Puaprasert, K., *et al.* Real time PCR detection of common CYP2D6 genetic variants and its application in a Karen population study. *Malar J* 2018;17(1):427.
2. https://genome.med.harvard.edu/documents/sequencing/Agencourt_AMPure_Protocol.pdf
3. http://tools.thermofisher.com/content/sfs/manuals/qubit_assays_qrc.pdf

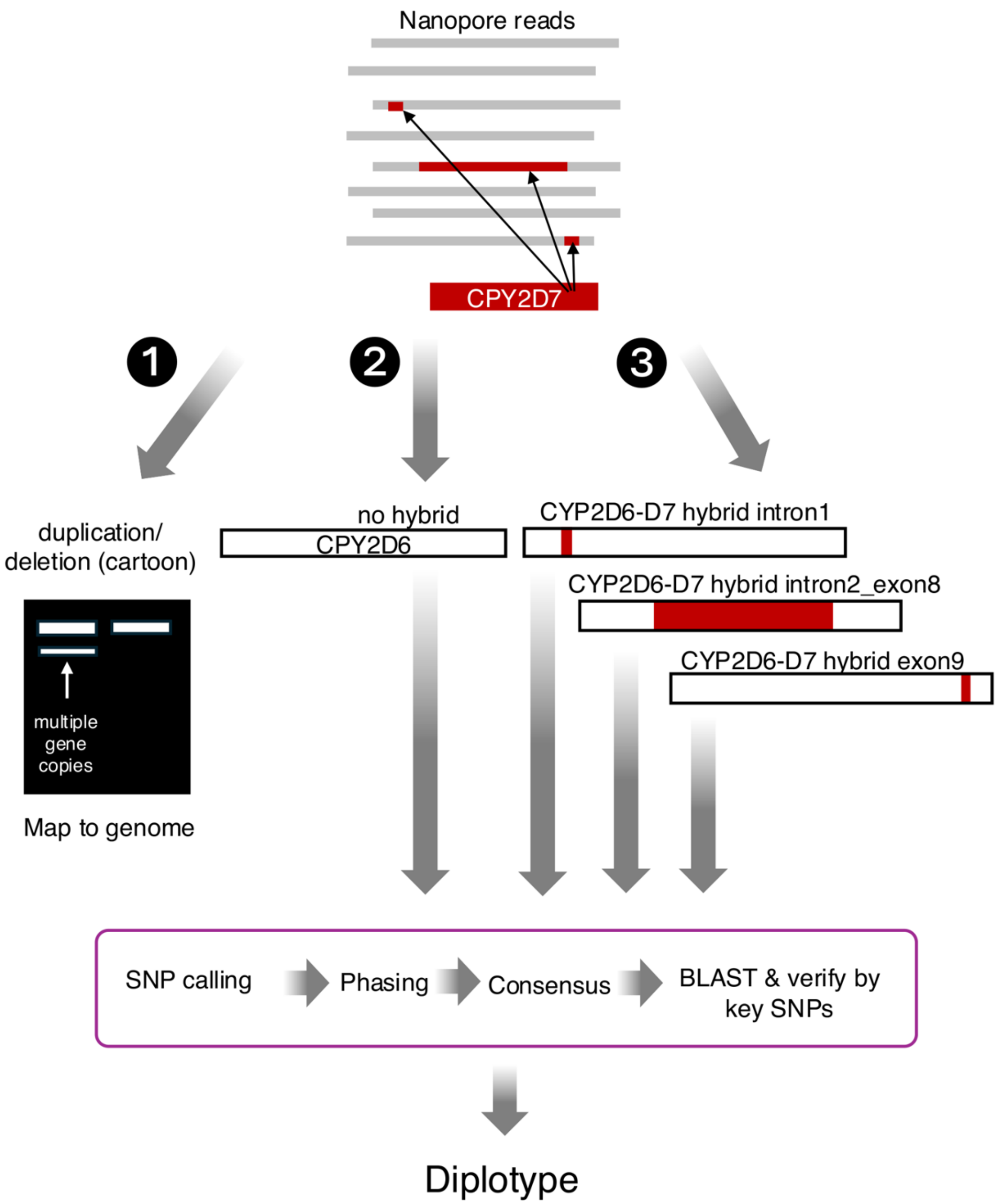

Supplementary Figure 1. Workflow for CYP2D6 Diplotype Assignment.
Nanopore sequencing reads were mapped to the human reference genome (hg19). Step 1 involved identifying duplications and deletions based on mapped reads outside of CYP2D6. Steps 2-3 classified reads into four groups: 1) no hybrid (no_hybrid), 2) CYP2D6-D7 hybrid at intron 1 (hybrid_intron1), 3) CYP2D6-D7 hybrid between intron 2 and exon 8 (hybrid_intron2_exon8), and 4) CYP2D6-D7 hybrid at exon 9 (hybrid_exon9). Finally, SNP calling, phasing, consensus sequence generation, and BLAST verification of key SNPs were performed to assign the final CYP2D6 diplotype, providing a comprehensive analysis of its functional status.

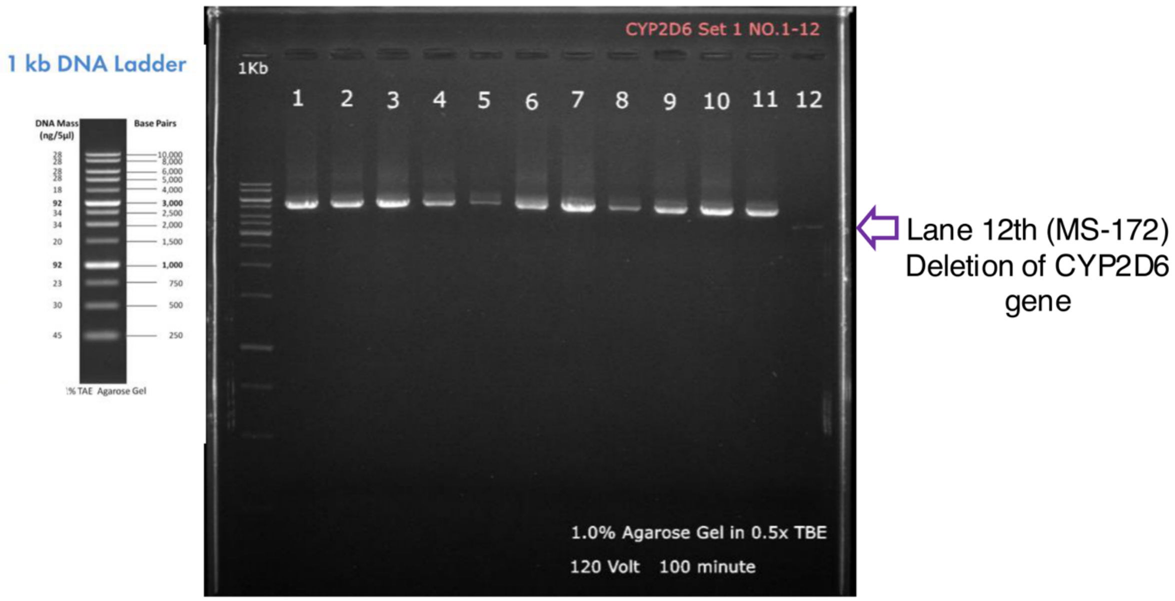

Supplementary Figure 2. PCR Validation of CYP2D6 Gene Amplification in Clinical Samples.

PCR amplification of 12 clinical samples (ordered according to Table 2) confirmed the expected amplicon size of 5.1 kb, validating the optimized PCR conditions for clinical DNA. Lane 12th corresponds to sample MS-172, which did not produce an amplicon for CYP2D6 gene copies but displayed a faint band at 3.5 kb (indicated by the purple arrow), suggesting a structural variation or deletion in the CYP2D6 region.

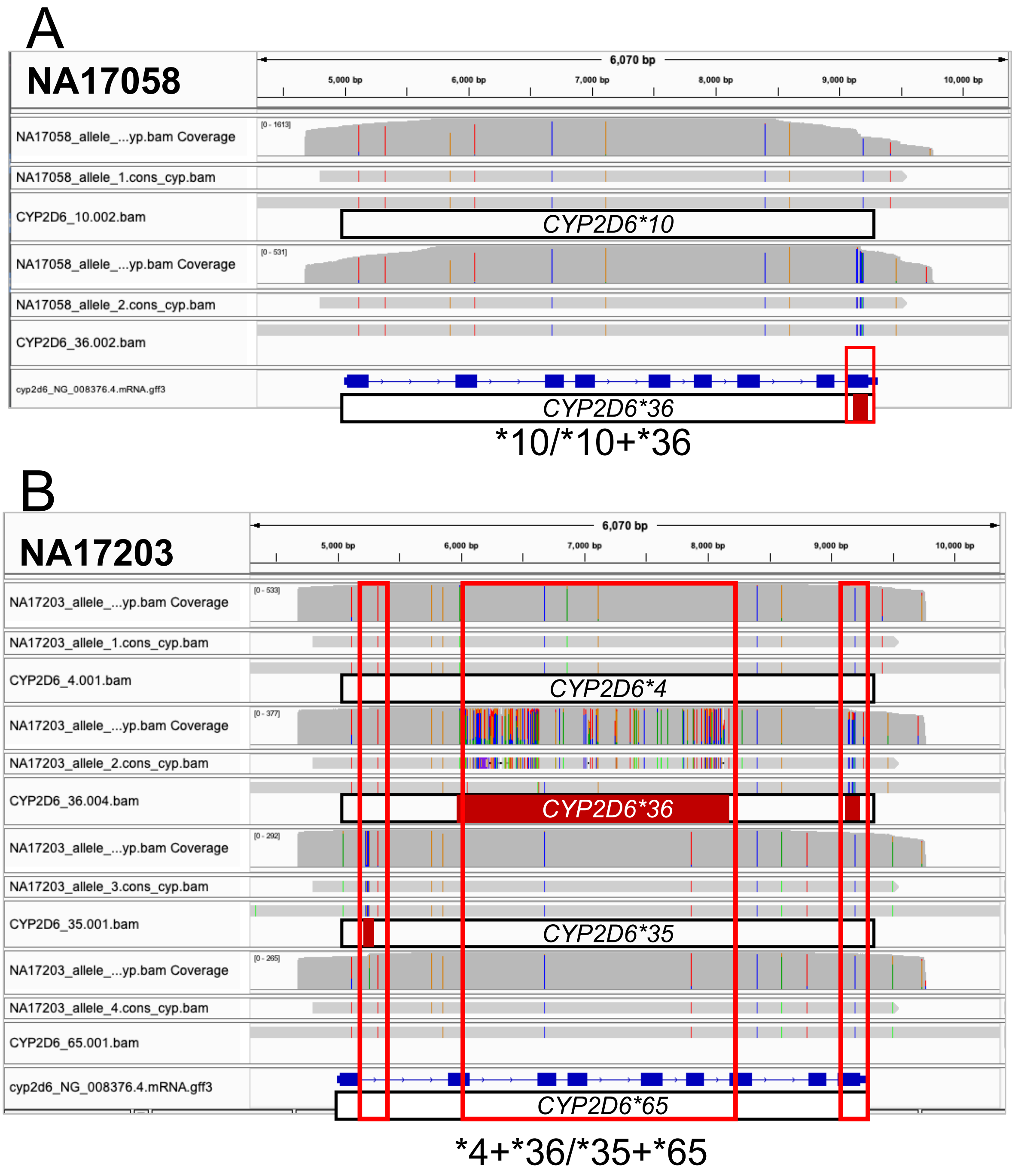

Supplementary Figure 3. Visualization of CYP2D6-D7 Hybrids in DNA Standard Samples.
IGV plots display CYP2D6-D7 hybrid structures in DNA standard samples NA17058 (A) and NA17203 (B), containing two and four alleles, respectively. Each allele is represented with tracks showing read coverage, consensus sequence, and reference allele annotated with star (*) allele designations. These visualizations confirm hybrid configurations and the incorporation of CYP2D7 gene segments, consistent with prior reports.

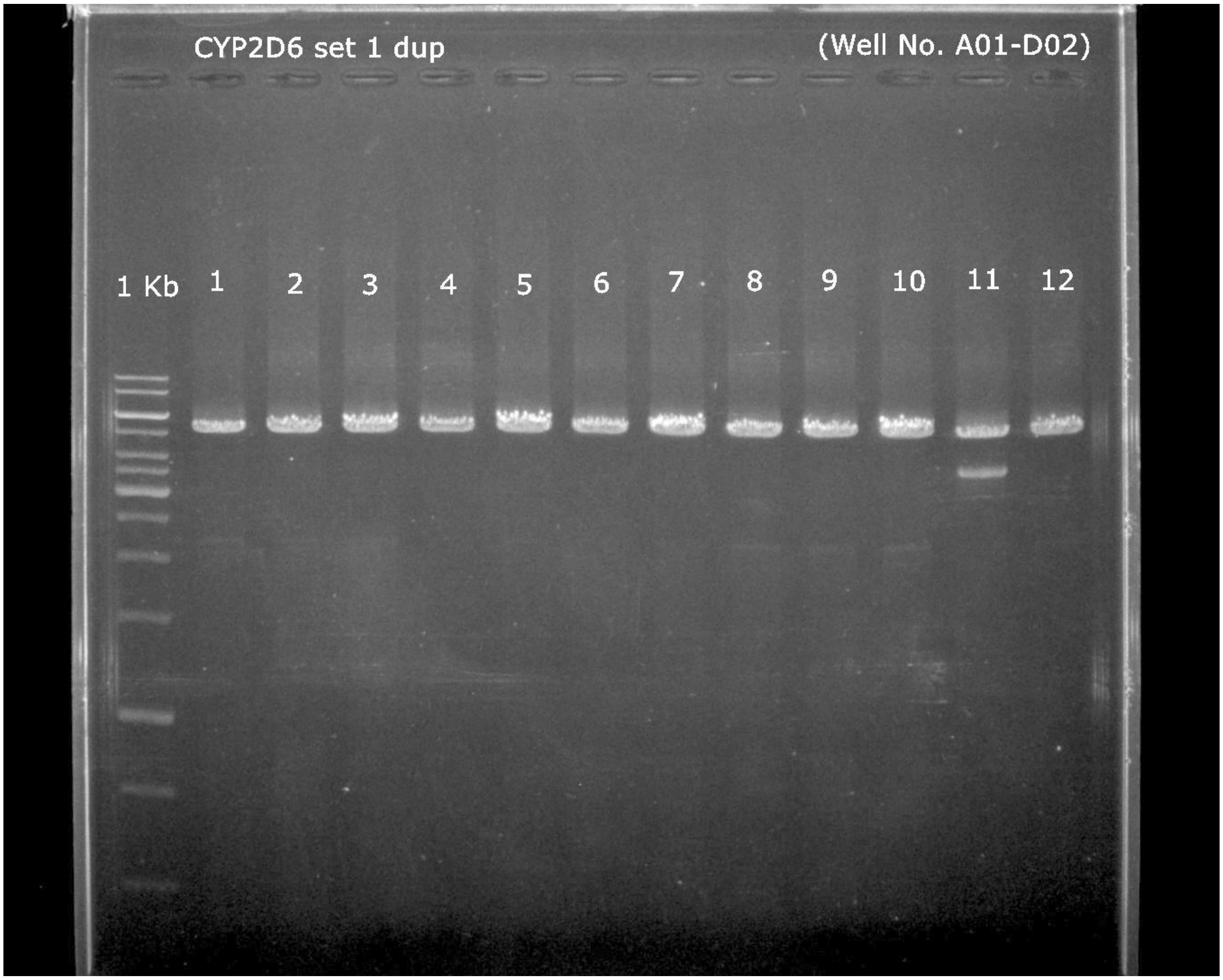

Supplementary Figure 4A. The original images of agarose gel electrophoresis analysis for the *CYP2D6* gene and *CYP2D6* gene duplication in samples No. 1-12.

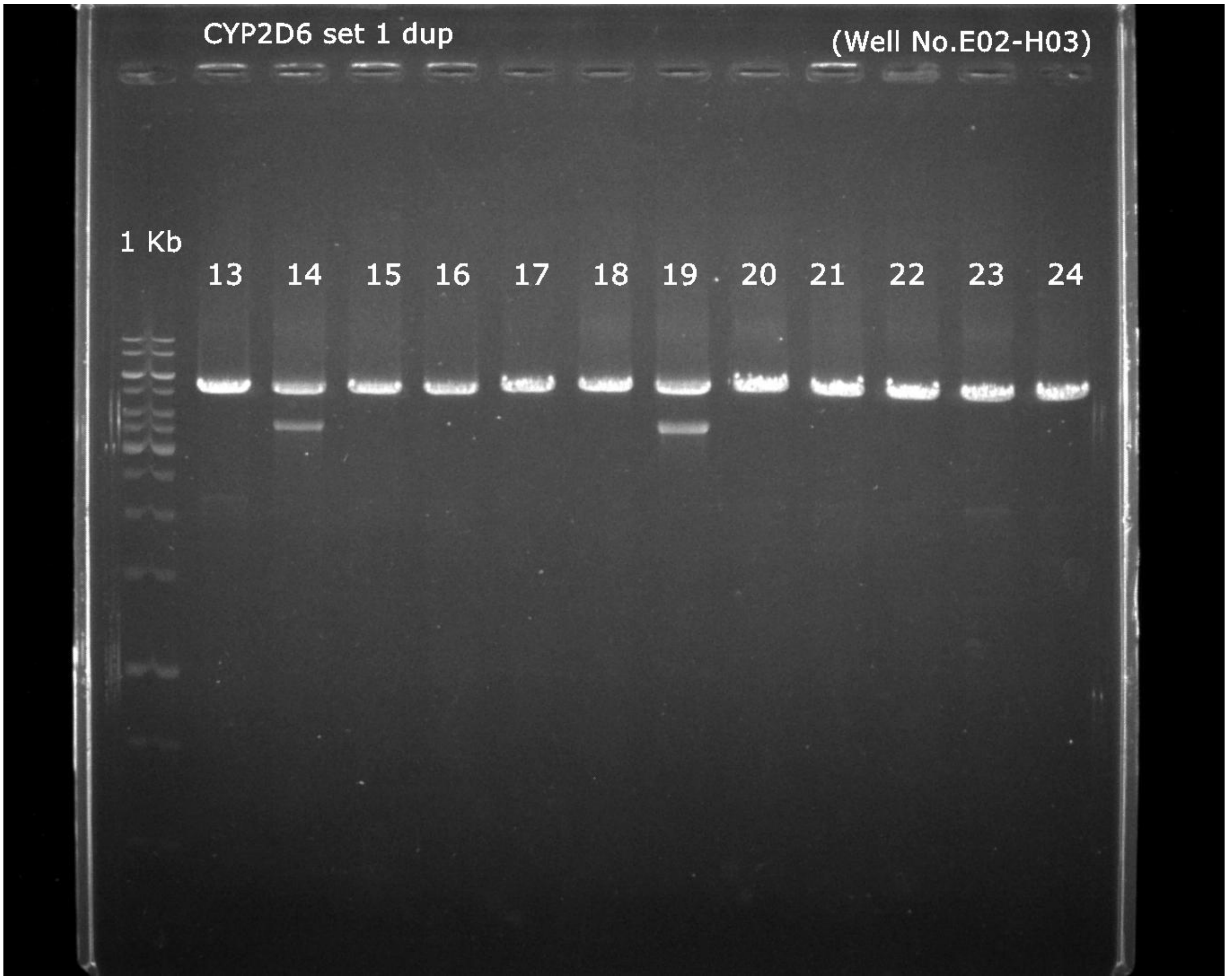

Supplementary Figure 4B. The original images of agarose gel electrophoresis analysis for the *CYP2D6* gene and *CYP2D6* gene duplication in samples No. 13-24.

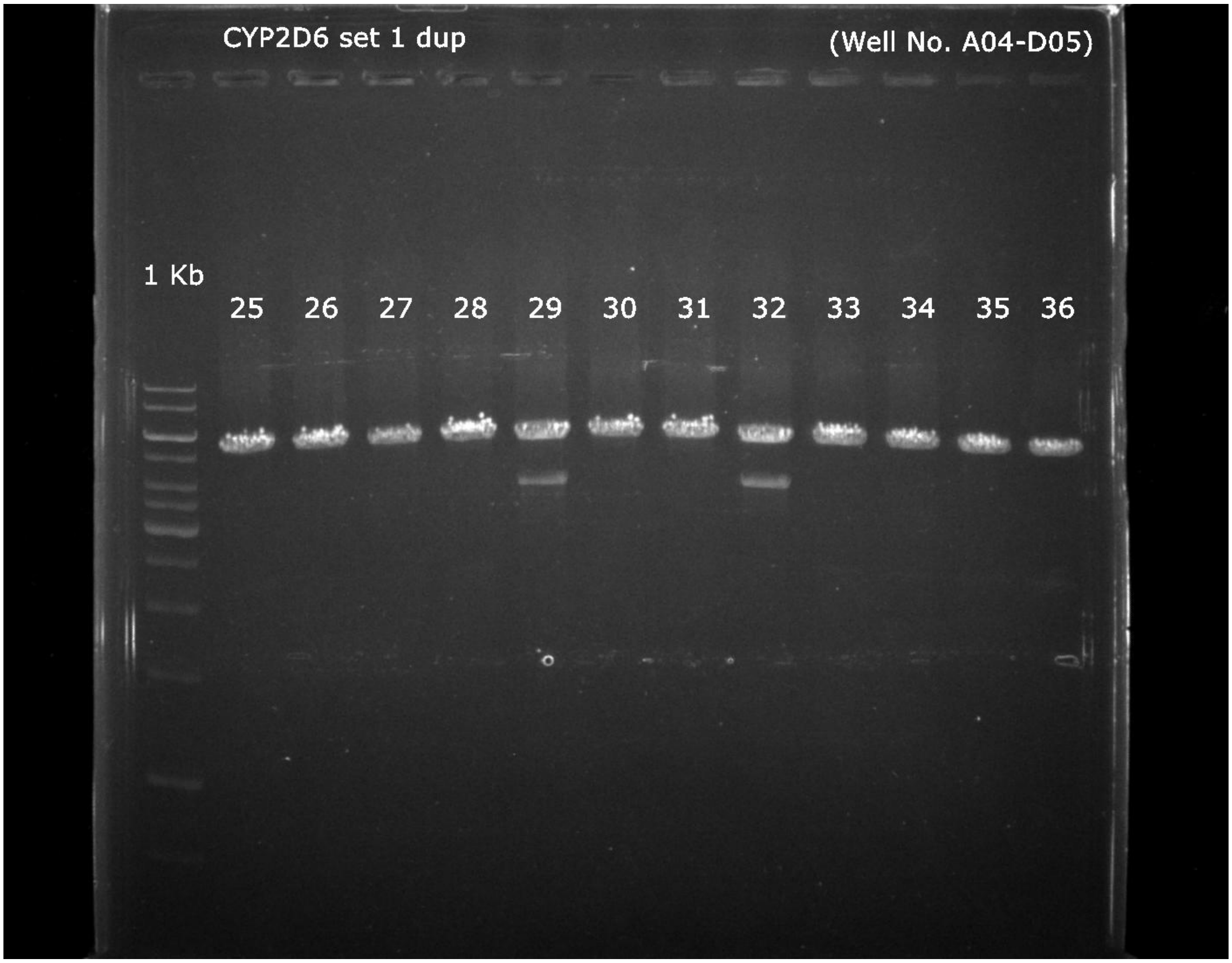

Supplementary Figure 4C. The original images of agarose gel electrophoresis analysis for the *CYP2D6* gene and *CYP2D6* gene duplication in samples No. 25-36.

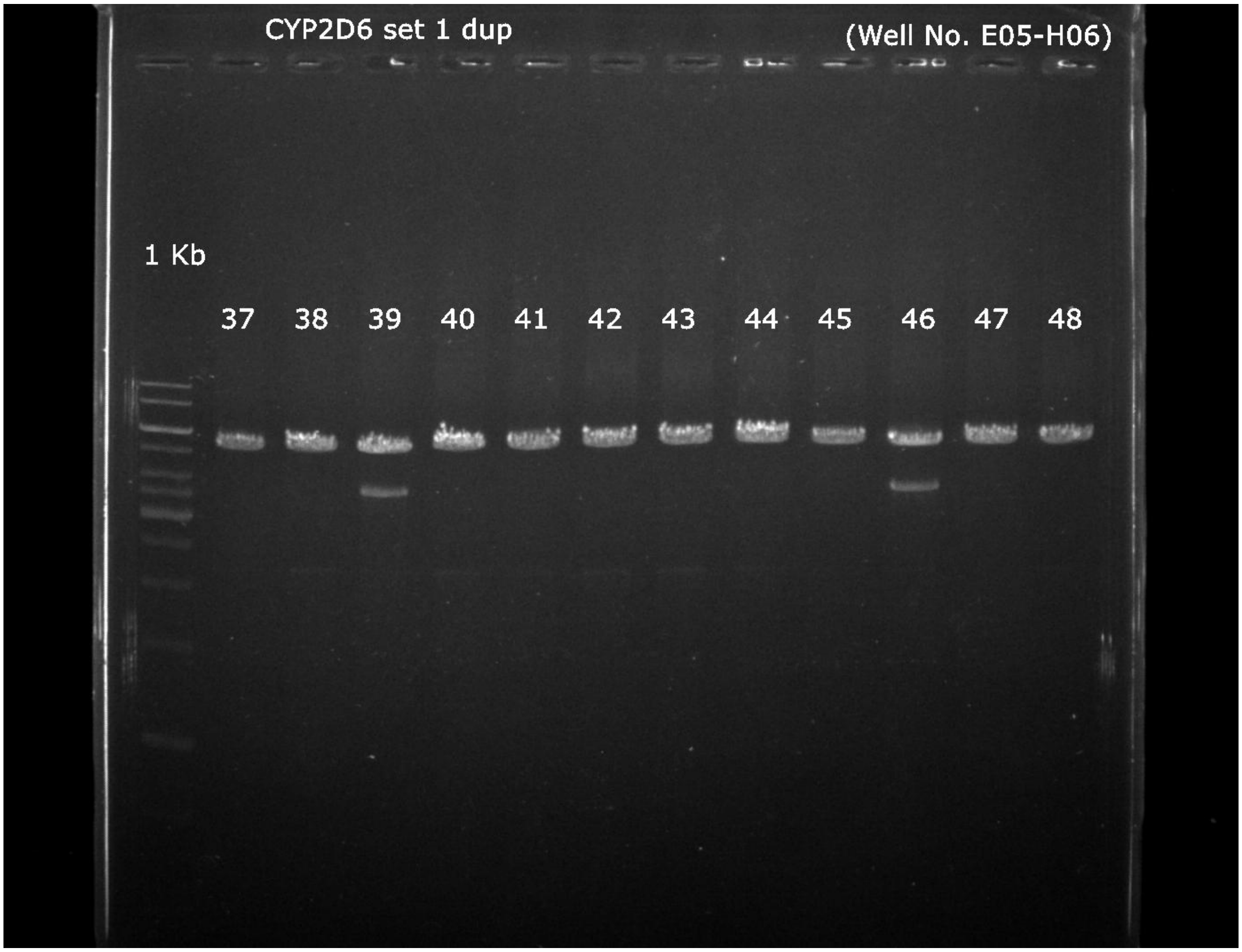

Supplementary Figure 4D. The original images of agarose gel electrophoresis analysis for the *CYP2D6* gene and *CYP2D6* gene duplication in samples No. 37-48.

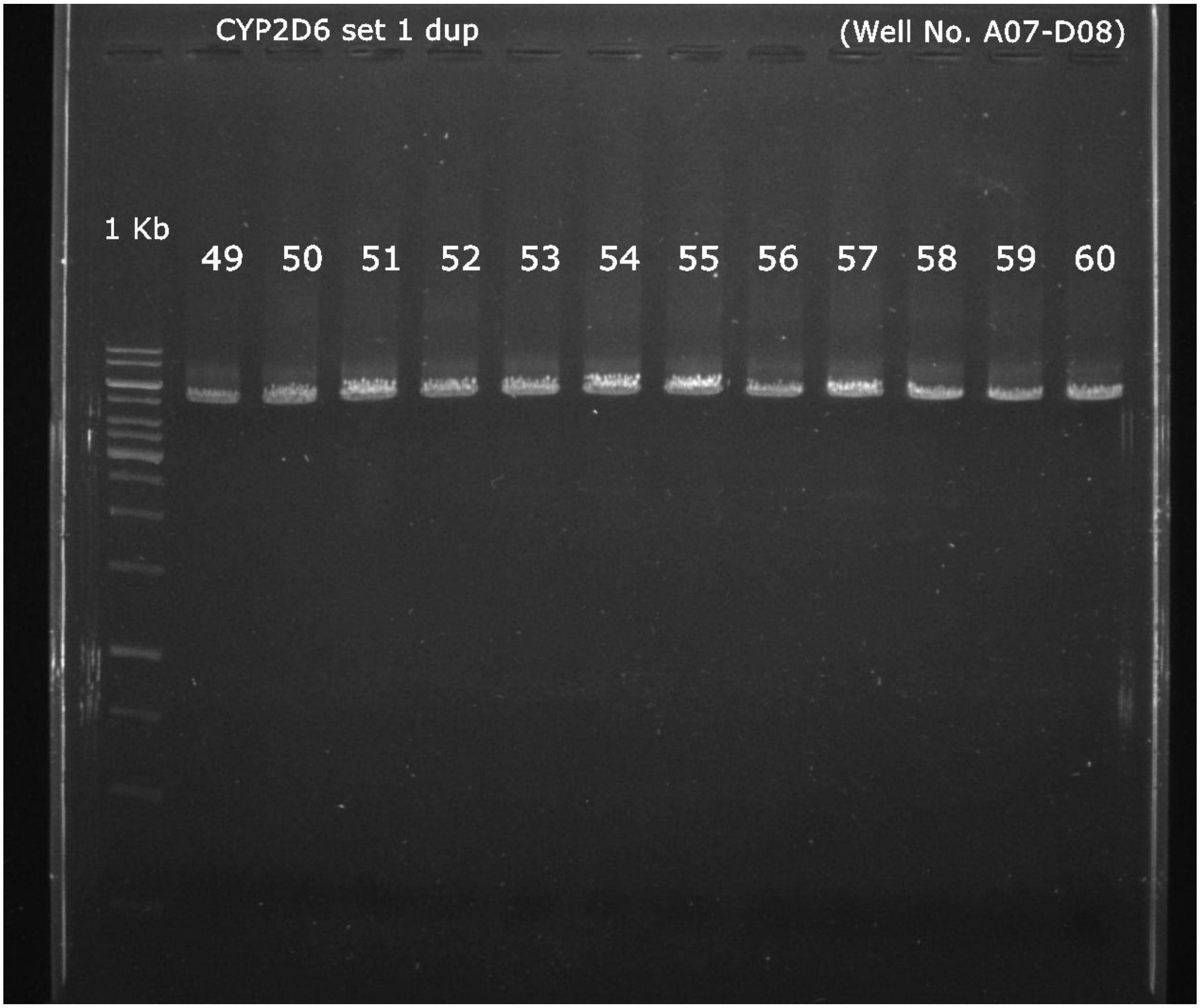

Supplementary Figure 4E. The original images of agarose gel electrophoresis analysis for the *CYP2D6* gene and *CYP2D6* gene duplication in samples No. 49-60.

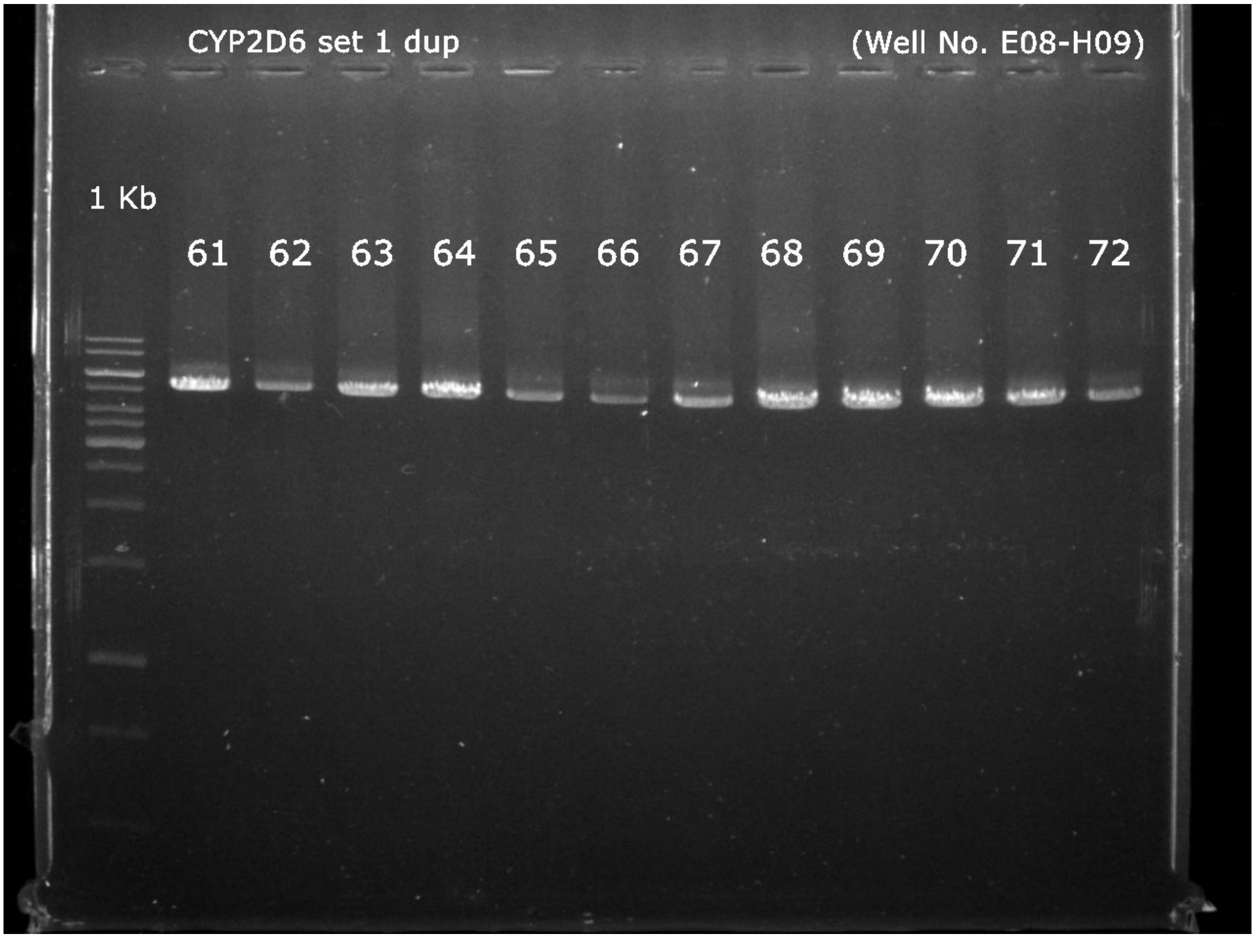

Supplementary Figure 4F. The original images of agarose gel electrophoresis analysis for the *CYP2D6* gene and *CYP2D6* gene duplication in samples No. 61-72.

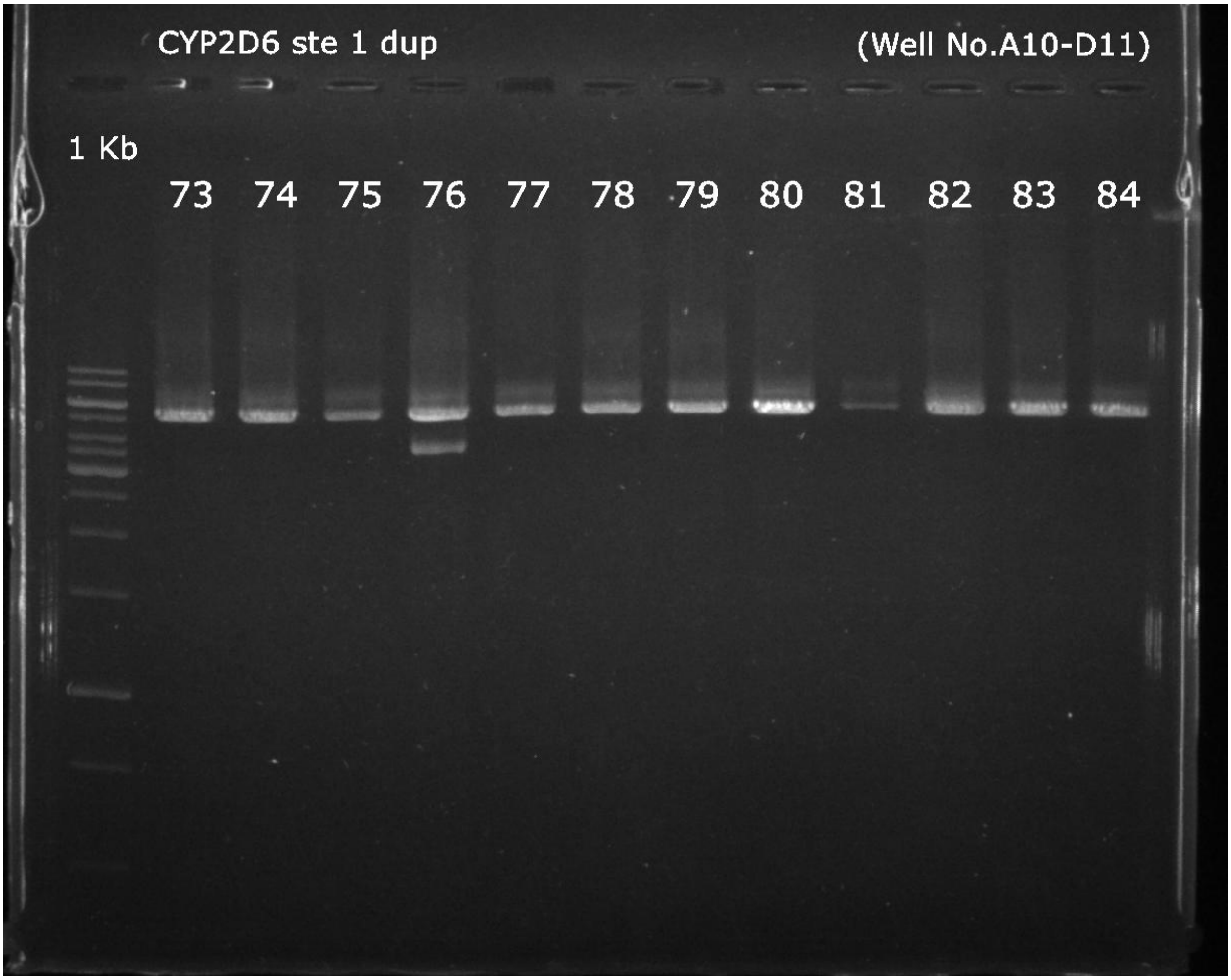

Supplementary Figure 4G. The original images of agarose gel electrophoresis analysis for the *CYP2D6* gene and *CYP2D6* gene duplication in samples No. 73-84.

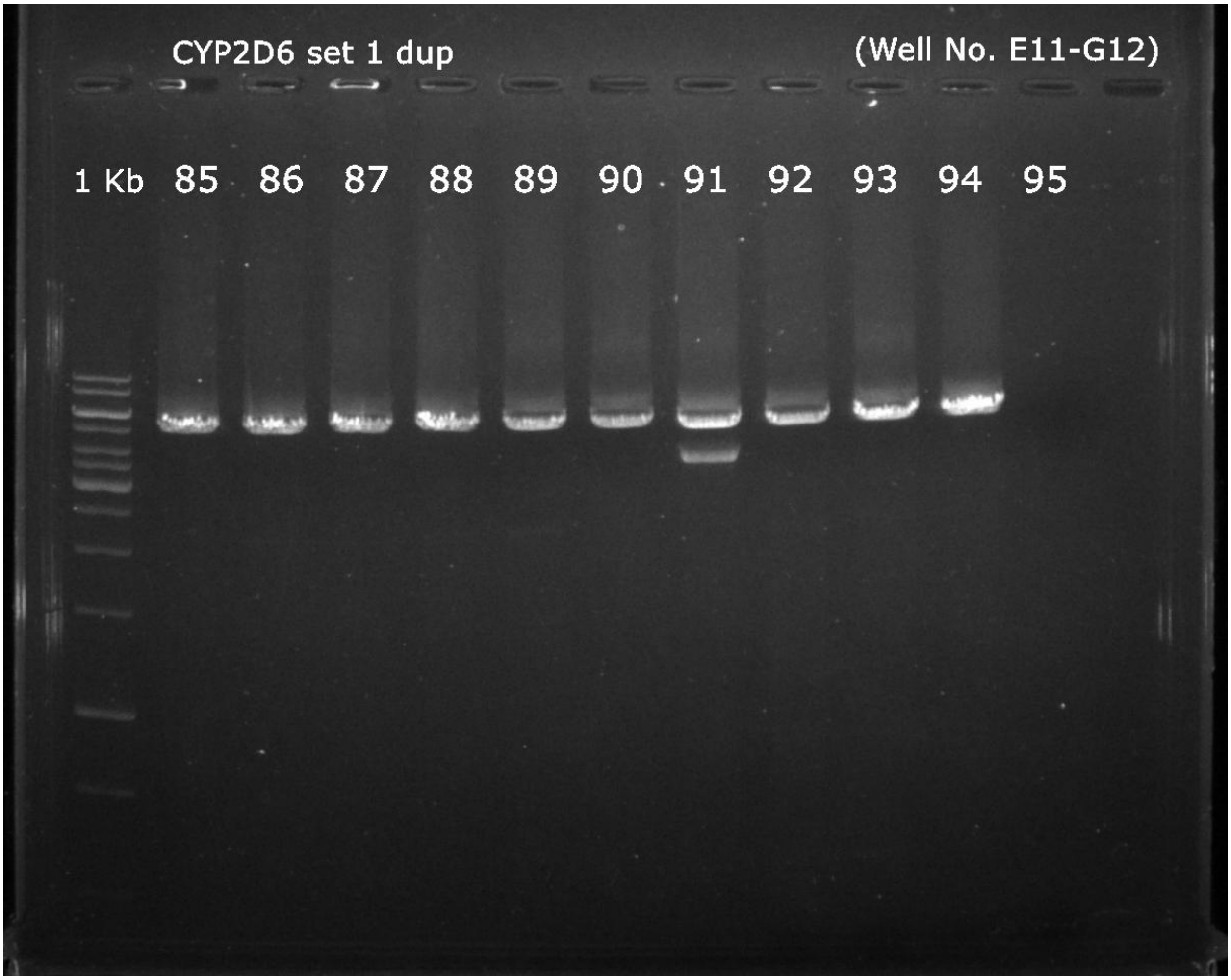

Supplementary Figure 4H. The original images of agarose gel electrophoresis analysis for the *CYP2D6* gene and *CYP2D6* gene duplication in samples No. 85-95.
